# Exosomal miR-30a-5p promoted intrahepatic cholangiocarcinoma progression by increasing angiogenesis and vascular permeability in PDCD10 dependent manner

**DOI:** 10.7150/ijbs.83170

**Published:** 2023-08-28

**Authors:** Wangjie Jiang, Xiaoli Shi, Lizhu Sun, Yaodong Zhang, Xiangxu Kong, Xiao Yang, Yongmei Yin, Changxian Li, Xiangcheng Li

**Affiliations:** 1 Hepatobiliary Center, The First Affiliated Hospital of Nanjing Medical University, Nanjing, Jiangsu Province, China.; 2 Key Laboratory of Liver Transplantation, Chinese Academy of Medical Sciences; NHC Key Laboratory of Living Donor Liver Transplantation (Nanjing Medical University), Nanjing, Jiangsu Province, China.; 3 School of Medicine, Southeast University, Nanjing, Jiangsu Province, China.; 4 Department of Oncology, The First Affiliated Hospital of Nanjing Medical University, Nanjing, Jiangsu Province, China.

**Keywords:** cholangiocarcinoma, miR-30a-5p, PDCD10, angiogenesis, permeability

## Abstract

Tumor-associated angiogenesis positively associates with malignant metastasis of intrahepatic cholangiocarcinoma (ICCA). Cancer cell-derived exosomes carrying microRNAs involves in tumor microenvironment (TME) regulation. We aimed to evaluate exosomal miR-30a-5p in ICCA development. Our data showed that increased miR-30a-5p level was correlated with higher microvascular density (MVD) and worse prognosis. Augmented miR-30a-5p expression was induced by hypoxia induced factor 1α (HIF-1α) in ICCA cell. Further exploration revealed that ICCA-derived miR-30a-5p could be transferred to endothelial and increased endothelial cells recruitment and proliferation, induced angiogenesis and vascular permeability in exosome dependent manner. In addition, circulating exosomal miR-30a-5p was higher in ICCA patients, and correlated with ICCA tissues-expressing miR-30a-5p. Hypoxic stress enhanced the effects of exosomal miR-30a-5p on endothelial-associated phenotypes. Rescued experiments showed that exosomal miR-30a-5p modulated endothelial-associated phenotypes in a way relied on programmed cell death 10 (PDCD10). Moreover, we revealed that the packing of miR-30a-5p into ICCA cells-derived exosomes was mediated by eukaryotic translation initiation factor 4B (EIF4B). More importantly, the combined application of targeting miR-30a-5p and apatinib could synergistically improve antiangiogenic efficacy in ICCA. Combined, ICCA-derived exosomal miR-30a-5p could be an excellent therapeutic and monitoring indicator for ICCA patients.

## Introduction

Being one of the highly lethal malignancies that accounts for 3% of gastrointestinal cancers, cholangiocarcinoma (CCA) obtains global attention due to a notable uptrend of its incidence and mortality in recent decades, with an evident increase in specific regions such as China, South Korea and Thailand 1. As a subtype of CCA derived from the second order bile ducts 2, intrahepatic cholangiocarcinoma (ICCA) is featured by invasive growth and earlier lymphatic metastasis 3. Despite tremendous advances have been made in diagnosis and treatment of ICCA, the inability to detect earlier and prevent postoperative recurrence remained a giant challenge, with current 5-year survival less than 20% 1. Therefore, detailed studies for further understanding the events of ICCA tumorigenesis and metastasis are urgently needed.

Tumor microenvironment (TME), constituted of multiple cell populations and complex matrix, is typical of poorly differentiated vasculature, lack of oxygen and nutrient, and uncontrolled growth of cancer cells. TME has been reported to be involved in cancer progression and therapeutic resistance 4, 5. As a typical hallmark of TME, hypoxia in many solid malignancies directly induces the expression of hypoxia-inducible factor 1-alpha (HIF-1α) which then stimulates its downstream molecules including vascular-endothelial growth factors (VEGFs), contributing to new vasculature and angiogenesis 6. However, the tumor-induced vasculature is characterized by immaturity of vessels and lack of mural cells, causing increased vascular permeability and enhanced hematogenous metastasis of cancer cells 7. ICCA, however, presents in hypovascularized and hypoxic TME which prompts the production of higher pro-angiogenic factors such as VEGFs and subsequent tube formation in stroma 8, 9. In addition, hypoxic TME has reported to be associated with exosomes-mediated intercellular communication 10, 11.

Deriving from multivesicular bodies (MVBs), exosomes are a subpopulation of extracellular vehicles (EVs) with the size in 30-150 nm diameter. Initially, exosomes were considered to be cellular waste loading useless cellular components or metabolites 12. With the further research progress and deeper understanding, exosomes now are deemed as a novel regulator for intercellular communication mediating by transferring loaded proteins, glycans and nucleic acids to recipient cells, thus causing the alteration of biological behaviors 13. For instance, colorectal cancer-secreted exosomes carrying miR-934 transferred to macrophage and induced immunosuppressive TME, thus facilitating liver metastasis 14. Exosomal miR-1247-3p produced by hepatocellular carcinoma (HCC) cells facilitated activation of cancer-associated fibroblasts (CAFs) which then foster lung metastasis 15. Likewise, CAF-derived exosomes could enhance cancer cells survival and induce chemoresistance in gastric cancer and pancreatic cancer 16, 17. We previously demonstrated that miR-30a-5p in CCA acts as an oncogene augmenting cell growth and suppressing cell apoptosis 18. However, whether miR-30a-5p could be transferred via exosomes and involved in CCA intercellular communication remained not investigated.

In the study, we intended to investigate the role of exosomal miR-30a-5p derived from ICCA cells in angiogenesis. We revealed that hypoxia-induced HIF-1α elevated the level of miR-30a-5p of which upregulation in ICCA tissues was positive correlated with more MVD and worse survival. ICCA cells-released exosomes harboring miR-30a-5p induced tumor-associated angiogenesis and elevated endothelium permeability, ultimately facilitating invasive behavior of cancer cells to endothelium and subsequent hematogenous metastasis by targeted PDCD10. In addition, combined use of anti-angiogenesis and targeting miR-30a-5p therapy might be more effective for ICCA patients. Our findings unveil that miR-30a-5p might be an excellently blood-based biomarker and therapeutic target for ICCA patients.

## Materials and methods

### ICCA patients and tissue microarray (TMA)

ICCA patients underwent surgical procedures from June, 2008 to May, 2017 at the First Affiliated Hospital of Nanjing Medical University, Nanjing China, were included in the present study. Inclusion criteria: (i) Two independent pathologists confirmed the tissues as ICCA; (ii) None of the patients underwent radiotherapy, chemotherapy or other related therapy before surgery. Exclusion criteria: (i) ICCA patients received related therapy before surgery; (ii) ICCA patients with other malignant diseases.

Immunohistochemistry (IHC) staining and in situ hybridization in TMA including 114 CCA patients was performed to validate the clinical significance of angiogenesis and miR-30a-5p. The quantitation of immunostaining for CD31 and in situ hybridization for miR-30a-5p was determined as previously described^19^. Briefly, the score of the positive rates were recorded as 0 point (0-5%), 1 point (6-35%), 2 points (36-70%), 3 points (71-100%). The staining intensity score was presented as 0 point (none), 1 point (low), 2 points (medium), and 3 points (high). The total Q-score was the product of heterogeneity and intensity. The Q-score >4 was defined as high expression.

### Statistical analysis

Statistical analyses were carried out using GraphPad Prism 7. Each experiment was repeated at least three times with at least three replicates each time and data were presented as the mean ± standard deviation (SD). Normality test was checked before data analysis. Differences between groups were analyzed by Student's t test. Pearson correlation analysis was utilized to determine linear correlations. Survival analysis was performed based on Kaplan-Meier method and log-rank test. We deemed *P* < 0.05 as significant differences.

More materials and methods are provided in the Supporting information.

## Results

### MiR-30a-5p in ICCA correlates with high MVD and worse prognosis

**Figure 1:**
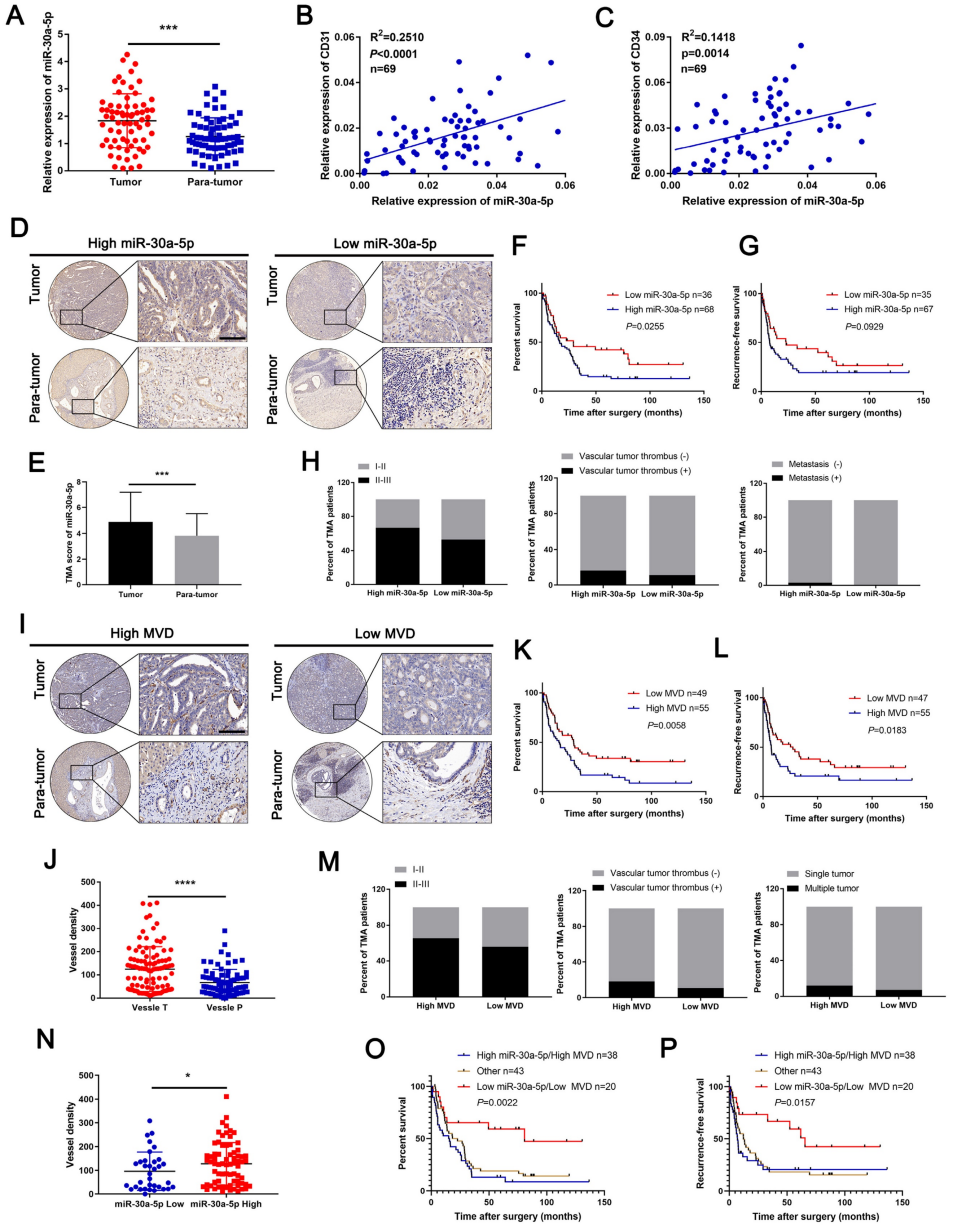
** Upregulated miR-30a-5p in ICCA induces high MVD and is correlated with worse prognosis. A** miR-30a-5p expression level was detected by qRT-PCR in sixty-nine pairs of CCA and paired adjacent tissues. **B, C** Correlation analysis between miR-30a-5p and CD31, CD34 was performed in sixty-nine patients based on qRT-PCR. **D, E** Representative images of distinct intensity of miR-30a-5p expression based on in situ hybridization (ISH) and the score of high/low miR-30a-5p level in tumor and their corresponding para-tumor tissues were shown, the scores were calculated by intensity and percentage of stained cells. **F, G** Kaplan-Meier curves for overall survival (OS) and recurrence-free survival (RFS) of ICCA patients with high/low miR-30a-5p expression level. **H** The percentage of ICCA patients with tumor stage, vascular tumor thrombus and metastasis or not in high/low miR-30a-5p was shown. **I** Representative images of MVDs based on CD31 IHC staining in tumor and their corresponding para-tumor tissues were shown. **J** The MVDs in tumor and their corresponding para-tumor tissues were shown. **K, L** Kaplan-Meier curves for OS and RFS of ICCA patients with high/low MVDs. **M** The percentage of ICCA patients with tumor stage, vascular tumor thrombus and intrahepatic metastasis or not in high/low MVD was shown. **N** The MVDs in tumor tissues with low or high miR-30a-5p were shown. Kaplan-Meier curves analysis for** O** OS and **P** RFS of ICCA patients combined with miR-30a-5p and MVD. *P < 0.05, **P < 0.01, ***P < 0.001.

Previously, we confirmed miR-30a-5p as an oncogene that promotes CCA cell proliferation. Further probation of TCGA database showed that miR-30a-5p could be an effective biomarker in distinguishing cancer and normal tissues (Figure S1A, 1B), which hints that miR-30a-5p could be a critical biomarker in CCA. However, whether miR-30a-5p could be involved in other malignant phenotypes of CCA remains poorly characterized. By expanding the sample size, we confirmed the apparently increased level of miR-30a-5p in 69 ICCA tissues (Figure 1A). In addition, a remarkably positive correlation was observed between miR-30a-5p and CD31, and between miR-30a-5p and CD34 (Figure 1B, 1C), suggesting that miR-30a-5p might be involved in cancer-associated angiogenesis.

To further unveil the relationship between miR-30a-5p and cancer-associated angiogenesis in ICCA, in situ hybridization (ISH) for miR-30a-5p and IHC for CD31 were performed in ICCA TMA. Based on the data, we detected upregulated miR-30a-5p level in ICCA tissues as compared to paired non-cancerous tissues and was mainly localized in tumor tissues (Figure 1D, 1E). Kaplan-Meier survival analysis showed that high-miR-30a-5p ICCA patients had worse overall survival (OS, *P*=0.0255) and recurrence-free survival (RFS, *P*=0.0929) (Figure 1F, 1G). Further exploration of the clinical parameters on the basis of TMA indicated that ICCA patients with higher miR-30a-5p level was more prone to be advanced stage, vascular invasion and metastasis (Figure 1H). Subsequently, TMA analysis for MVD revealed that high-MVD was positively correlated with worse OS (*P*=0.0058) and RFS (*P*=0.0183) for ICCA patients (Figure 1I-1L). Clinical parameters analysis showed that high-MVD ICCA patients were more likely to be present with advanced stage, vascular tumor and multiple intrahepatic foci (Figure 1M). Additionally, we detected that high-miR-30a-5p ICCA patients tended to be accompanied by high MVD (Figure 1N). Further analysis showed that ICCA patients with low miR-30a-5p/low MVD apparently have better survival as compared to the others (Figure 1O, 1P).

Subsequent COX analysis indicated that among the risk factors, lymph node metastasis (HR: 2.883, 95% CI: 1.729-4.807) and miR-30a-5p expression level (HR: 1.676, 95% CI: 1.013-2.774) were independent predictive factors for postoperative OS (Table 1). Combined, the results suggested that miR-30a-5p might be a critical indicator for worse survival and inducing tumor-associated angiogenesis.

### HIF-1α induces miR-30a-5p expression in ICCA

**Figure 2:**
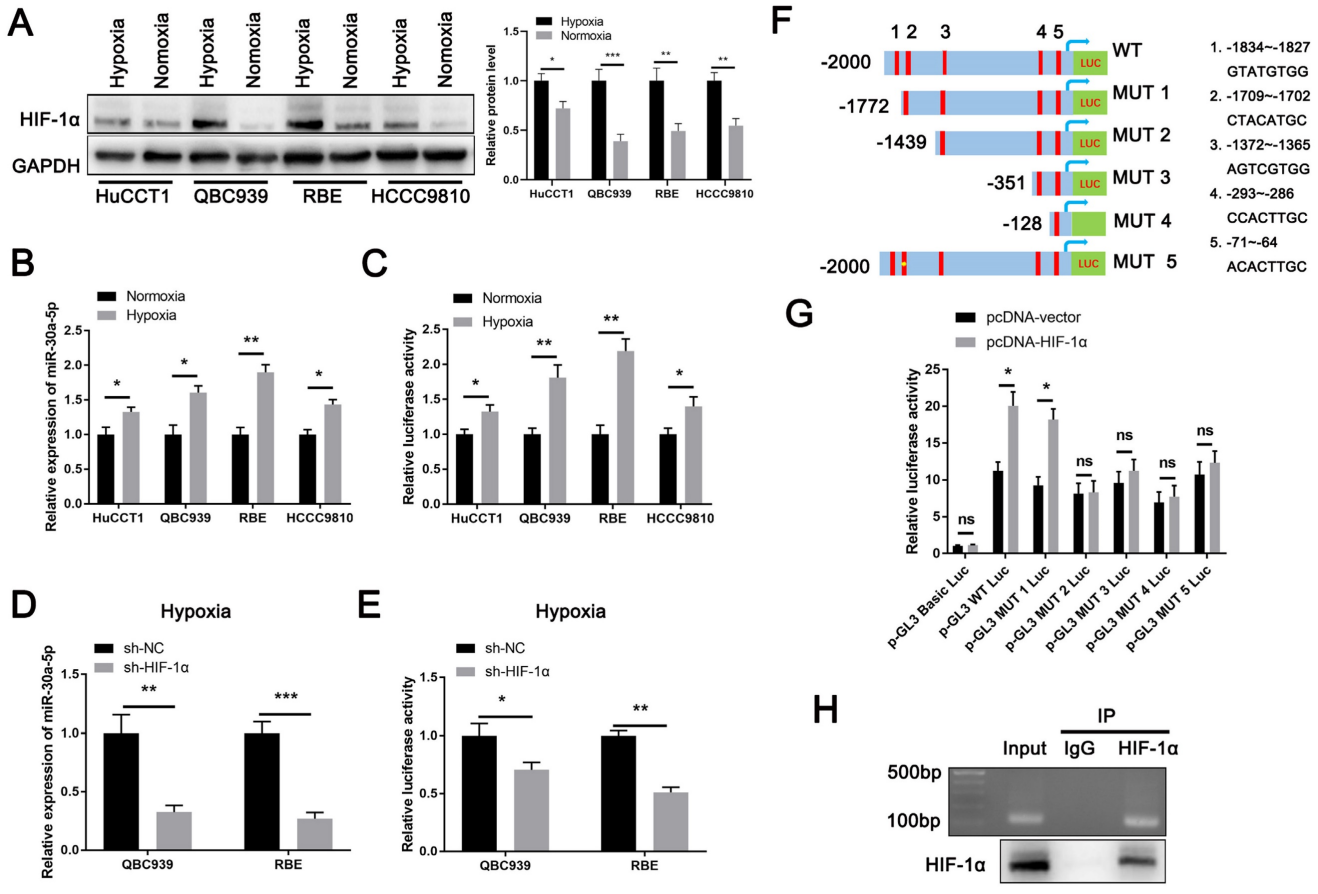
** HIF-1α induces miR-30a-5p expression in ICCA. A** The induced expression of HIF-1α in CCA cells under hypoxic and normoxic condition was explored. **B** The expression of miR-30a-5p in CCA cells under hypoxic and normoxic condition was explored. **C** The luciferase activity of miR-30a-5p promoter was detected in CCA cells subjected to hypoxia and normoxia. **D** QBC939 and RBE cells were transfected with siRNA targeted for HIF-1α, then the cells were treated with hypoxia. The relative expression level of miR-30a-5p was detected by qRT-PCR. **E** The luciferase activity of miR-30a-5p promoter was detected in CCA cells with HIF-1α knockdown in hypoxia. **F, G** 293T cells were transfected with different luciferase reporter vectors containing truncation mutants of miR-30a-5p promoter, and pc-DNA HIF-1α or empty vector, then the luciferase activity of miR-30a-5p promoter was detected. **H** Agarose electrophoresis for ChIP analysis of HIF-1α binding to the miR-30a-5p promoter. *P < 0.05, **P < 0.01, ***P < 0.001.

Lack of oxygen and nutrients is an important factor for tumor-induced angiogenesis. Being one of the most typical characteristics in ICCA 9, intra-tumorous hypoxia prompts the initiation of HIF-1α upregulation which has reported to involve in CCA malignant phenotypes and modulate miRNAs expression 20-23. Therefore, we hypothesized HIF-1α might be an upstream regulator of miR-30a-5p. Based on online JASPAR database (https://jaspar.genereg.net/genome-tracks/), we identified HIF-1α as a potential transcription factor for miR-30a-5p (Fig. S2A). To verify that, we first treated four CCA cell lines with hypoxia (1% O2) for 48h and detected that HIF-1α was greatly activated in cells under hypoxic condition as compared to cells under normoxia (Fig. 2A). Accordingly, we found that miR-30a-5p expression was significantly induced in cells with hypoxia treatment (Fig. 2B). Given that, we next performed luciferase reporter assay and detected that miR-30a-5p promoter (-2000~+100) activity was increased in CCA cells subjected to hypoxia treatment (Fig. 2C). To further confirm the result, we silenced HIF-1α in QBC939 and RBE cells which were subsequent subjected to hypoxia treatment. Similarly, HIF-1α depletion in CCA cells suppressed miR-30a-5p expression and decreased promoter activity (Fig. 2D, 2E). Since five putative HIF-1α-binding sites was predicted based on JASPAR database, we next set out to identify which site/sites were HIF-1α-responsive. 5' deletion and a site mutation of the miR-30a-5p promoter was constructed based on the HIF-1α-binding sites (Fig. 2F). Notably, enhanced luciferase activity was observed in WT and MUT1 miR-30a-5p promoter with HIF-1α plasmid transfection rather than in other mutants, suggesting site2 (-1709~-1702) might be the potentially specific binding site (Fig. 2G). Subsequently, we designed primers for site2. ChIP-PCR analysis indicated that HIF-1α directly bound to miR-30a-5p promoter region (Fig. 2H), which means HIF-1α transcriptionally induce miR-30a-5p expression in ICCA.

We next assessed the expression level of HIF-1α in ICCA and found that HIF-1α was strikingly increased (Fig. S2B-S2D). Besides, a significant positive correlation between the expression of HIF-1α and miR-30a-5p was observed (Fig. 2E). Together, our data implied that upregulated HIF-1α plays a critical role in augmenting miR-30a-5p level.

### ICCA-derived exosomes carry miR-30a-5p

To clarify how miR-30a-5p induce angiogenic process, we first probed the GEO data (GSE106817) and detected that miR-30a-5p could be expressed and stably existed in peripheral blood derived from multiple type of cancers patients (Fig. S3A). Likewise, serum miR-30a-5p in biliary tract cancer patients could be a predictive recurrence and prognosis-associated marker (GSE119892) (Fig. S3B). Further exploration showed that miR-30a-5p could be loaded in exosomes derived from various types of cells (EVmiRNA, http://bioinfo.life.hust.edu.cn/EVmiRNA/#!/) (Fig. S3C). Considered that, we speculated that ICCA cells-secreted exosomes harbors miR-30a-5p and involves in TME regulation.

Subsequently, we constructed miR-30a-5p-silencing RBE cells and miR-30a-5p-overexpressing HCCC9810 cells as previously described (Fig. S4A). We first incubated HuVECs with conditioned culture (CM) from miR-30a-5p-inhibitor/RBE, miR-30a-5p-mimic/HCCC9810 and their corresponding normal control cells. EDU, wound healing, transwell, tube formation and 3D sprouts assays showed that HuVECs were more likely to proliferate, migrate, form tube and sprouts in response to the CM from miR-30a-5p-mimic/HCCC9810. By contrast, HuVECs cultured with the CM from miR-30a-5p-inhibitor/RBE revealed impaired effects (Fig. S5A-S5E). Aortic ring assay showed that supernatant derived from miR-30a-5p-mimic/HCCC9810 enhanced angiogenesis in vivo, whereas treatment with supernatant collected from miR-30a-5p-inhibitor/RBE had the opposite effects (Fig. S5F). Vascular permeability plays a critical role in the exchange between vessels, tissues, and organs 24. Immunofluorescence analysis for tight junction related proteins containing ZO-1 and occludin in HuVECs showed that CM from miR-30a-5p-silencing RBE cells treatment increased integrity of endothelial barrier. By contrast, weakened integrity of endothelial barrier was detected in HuVECs treated with CM from miR-30a-5p-mimic/HCCC9810 cells (Fig. S5G, S5H), which was consistent with in vitro rhodamine-based permeability assay (Fig. S5I). Further IHC examination for CD34 in xenograft tumors originated from RBE cells with stable miR-30a-5p knockdown or not revealed that miR-30a-5p depletion evidently decreased the MVD in xenografts (Fig. S5J). In addition, less rhodamine infiltrated into xenografts removed from nude mice inoculated with miR-30a-5p-silencing RBE cells (Fig. S5K).

**Figure 3:**
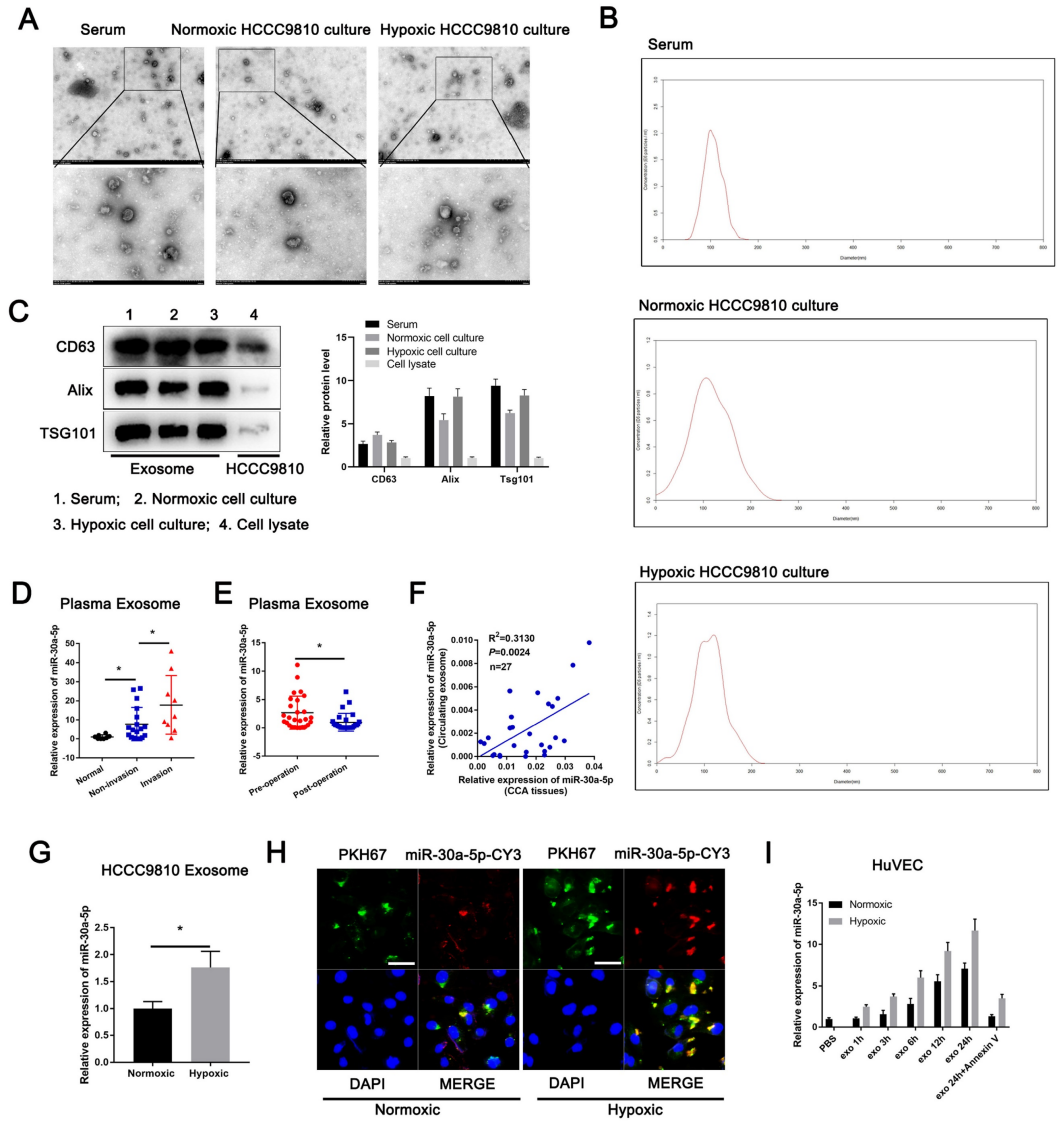
** Exosomal miR-30a-5p enhanced endothelium angiogenesis and permeability** Exosomes obtained from ICCA patients' peripheral blood, HCCC9810 cells subjected to hypoxic or normoxic condition were visualized by **A** transmission electron microscopy, **B** NTA analysis and **C** western blotting for CD63, Alix and TSG101. **D** Plasma-derived exosomal miR-30a-5p was examined in healthy individuals and ICCA patients with vascular invasion or not. **E** Plasma-derived exosomal miR-30a-5p in ICCA patients was determined and compared in pre-operation and post-operation (n=27). **F** The correlation between ICCA tissues-derived miR-30a-5p and circulating exosomal miR-30a-5p was analyzed (n=27). **G** HCCC9810 cells with miR-30a-5p-CY3 transfection were exposed to hypoxic or normoxic condition, then the HCCC9810-secreated exosomes were obtained and labeled with PKH67. Exosomal miR-30a-5p was detected based on qRT-PCR. **H** The representative images demonstrate the presence of CY3 fluorescence and PKH67 lipid dye in HUVECs after incubating PKH67 labeled exosomes with HuVECs for 24 hours. **I** The labeled exosomes or PBS were incubated with HuVECs for different times, then exosomal miR-30a-5p was detected by qRT-PCR. *P < 0.05, **P < 0.01, ***P < 0.001.

Then, we separated and purified exosomes from ICCA patients' serum and HCCC9810 cells in normoxia or hypoxia (Fig. 3A). The isolated exosomes were confirmed by NTA and western blotting of the marker including CD63, Alix and TSG101 (Fig. 3B, 3C). We detected miR-30a-5p expression level in normal and ICCA cases serum-derived exosomes, with a more evident level in ICCA patients with vascular invasion (Fig. 3D). Furthermore, higher circulating miR-30a-5p level in ICCA patients was notably decreased when patients underwent radical resection (Fig. 3E). More importantly, a significant correlation was observed between ICCA tissues-expressing miR-30a-5p and circulating exosomal miR-30a-5p (Fig. 3F), implying that peripheral blood-derived exosomal miR-30a-5p might be attributed to the high-expression of miR-30a-5p in ICCA tissues. Apart from that, miR-30a-5p level was much higher in HCCC9810-derived exosomes under hypoxic condition as compared to normoxia (Fig. 3G). We then obtained exosomes from medium in HCCC9810 cells with miR-30a-5p-CY3 overexpression under normoxic and hypoxic condition, and labeled these exosomes with PKH67 lipid dye. Given that miR-30a-5p level was involved in angiogenesis and vascular permeability, we thereby incubated the exosomes with HuVECs. We found that both miR-30a-5p-CY3 and PKH67 immunofluorescence were observed when HuVECs were subjected to incubate with these exosomes, with more evident trend in HuVECs incubating with hypoxic cells-derived exosomes (Fig. 3H). Consistently, miR-30a-5p expression level in HuVECs incubated with exosomes derived from normoxic and hypoxic HCCC9810 cells with miR-30a-5p-CY3 transfection was increased in time dependent manner. Hypoxia condition enhanced the effect. Moreover, treatment with Annexin V, an inhibitor of exosome internalization, significantly abolished the elevated miR-30a-5p level in HuVECs (Fig. 3I). Together, the results suggested that miR-30a-5p could be transferred from ICCA cell to endothelial via exosomes and induce tumor-associated angiogenesis and vascular permeability.

### ICCA-derived exosomes carrying miR-30a-5p induces angiogenesis and vascular permeability

**Figure 4:**
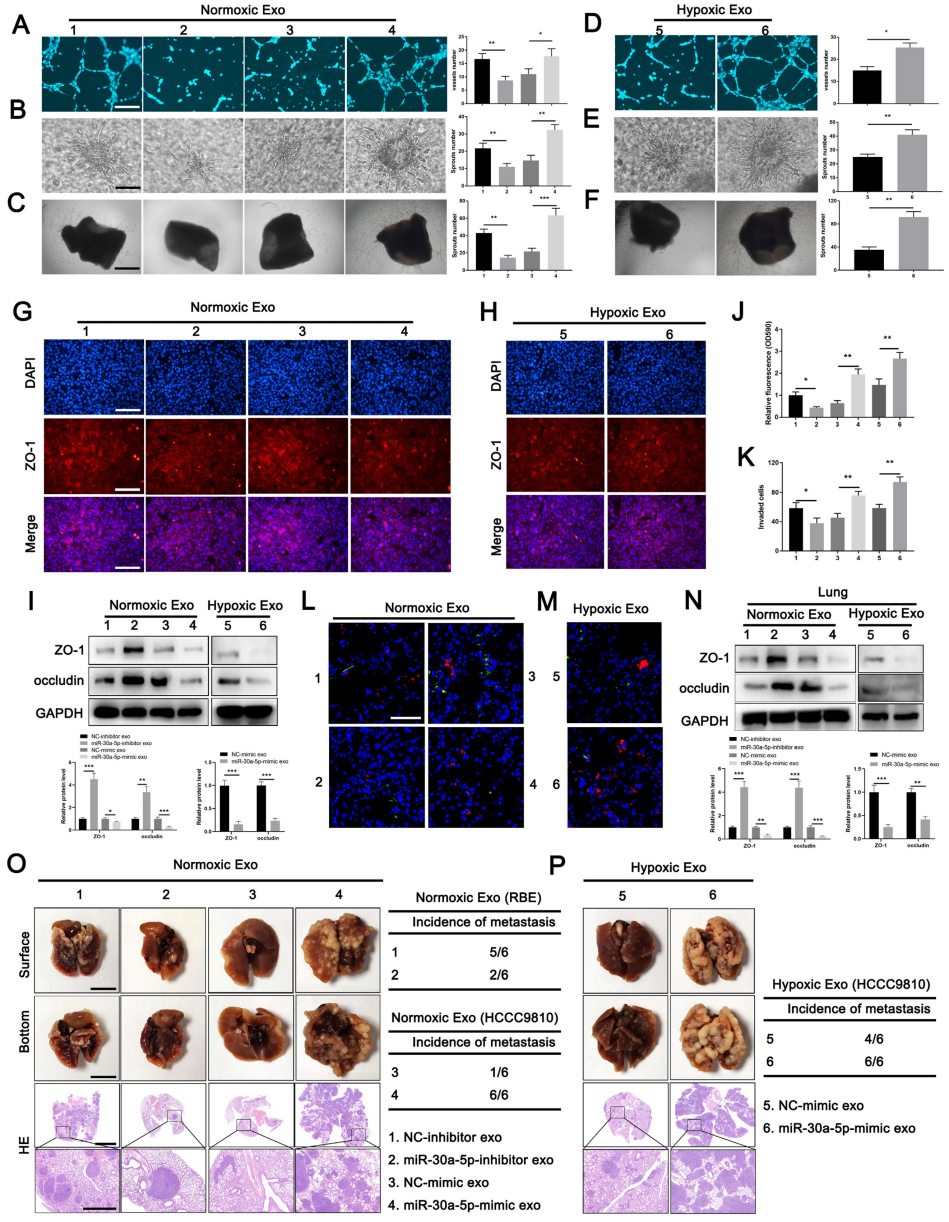
** Exosomes carrying miR-30a-5p induced angiogenesis and vascular permeability. A-C** Tube formation, 3D sprouts and aortic ring assays were performed to detect the effects of exosomes released from RBE/miR-30a-5p-inhibitor and normoxia-treated HCCC9810/miR-30a-5p-mimic on angiogenesis. **D-F** Tube formation, 3D sprouts and aortic ring assays were performed to detect the effects of hypoxia-treated HCCC9810/miR-30a-5p-mimic exosomes on angiogenesis. **G-I** The effects of normoxia/hypoxia-treated exosomes on endothelial permeability was explored by immunofluorescence and western blotting for tight junction related proteins including ZO-1 and occludin. **J** Permeability of the HUVECs monolayers to rhodamine, and **K** the ability of RBE cell to invade HuVECs monolayers after exposure to hypoxia/normoxia-treated exosomes are probed. **L, M** The mice were injected with rhodamine after exposure to PKH67-labeled exosomes. Then, the effects of hypoxia/normoxia-treated exosomes on vascular permeability of mice lung were examined via in vivo permeability assay. **N** The expression level of ZO-1 and occludin in lung of mice treated with hypoxia/normoxia-treated exosomes was detected by western blotting. **O, P** Mice were pretreated with 5 µg of hypoxia/normoxia-treated exosomes via tail vein every other day for 2 weeks, then mice were subjected to the injection of RBE (group 1 and 2) or HCCC9810 (group 3-6) cells. The representative images shown the metastatic foci in lung. *P < 0.05, **P < 0.01.

To determine the role of exosome-harboring miR-30a-5p in angiogenesis and vascular permeability, exosomes were extracted from RBE cells with miR-30a-5p knockdown, and HCCC9810 cells with miR-30a-5p mimic transfection under normoxic or hypoxic condition. We found the ability of HuVECs to form tube, sprouts in 3D model and aortic ring assay was impaired after treatment with miR-30a-5p-inhibitor/RBE-derived exosomes, but enhanced with miR-30a-5p-mimic/HCCC9810-derived exosomes treatment (Fig. 4A-4C). Hypoxia expose augmented the effect of exosomal miR-30a-5p on angiogenesis (Fig. 4D-4F). Then, HuVECs were subjected to the examination of integrity of endothelial barrier. Obviously, the integrity of ZO-1 and occludin were enhanced in HuVECs treated with exosomes released from RBE cells with miR-30a-5p silencing, but decreased after incubated by exosomes derived from miR-30a-5p-mimic/HCCC9810 cells. The regulatory effects were more evident under hypoxia condition (Fig. 4G-4I; Fig. S6A, 6B). Likewise, the same results were observed in rhodamine-based permeability experiment (Fig. 4J). Furthermore, more RBE cells invaded through single-layer HuVECs pretreated with exosomes extracted from miR-30a-5p-mimic/HCCC9810 cells under normoxic or hypoxic environment (Fig. 4K). These results hint that ICCA cell-secreted exosomes carrying miR-30a-5p might be involved in the regulation of endothelium permeability and subsequent cancer cell invasion, metastasis. To further examine the in vivo regulatory effects of exosomal miR-30a-5p in vascular permeability, exosomes derived from RBE and HCCC9810 cells under normoxia or hypoxia were labeled with PKH67, and then injected into mice via tail vein prior to rhodamine administration. Notably, exosomes from miR-30a-5p-suppressing RBE cells apparently decreased endothelium permeability of mice lung while opposite effect was detected in mice treated with exosomes from miR-30a-5p-overexpressing HCCC9810 cells, especially those from hypoxia-exposing HCCC9810 cells (Fig. 4L, 4M). Western blotting for ZO-1 and occludin in mice lung with exosomes treatment showed the same trend (Fig. 4N). However, little effect on biological characteristics of CCA cells subjected to the treatment of HuVECs/miR-30a-5p-mimic exosomes were observed (Fig. S7A-7C), suggesting that CCA cells-derived exosomes rather than endothelial-derived exosomes carrying miR-30a-5p played a critical role in regulating CCA TME.

To detect the effects of exosomal miR-30a-5p-induced vascular permeability on CCA metastasis, nude mice were intravenously pretreated with exosomes from RBE or HCCC9810 cells with silenced or overexpressed miR-30a-5p, and then were inoculated with RBE or HCCC9810 cells respectively. We detected mice treated with exosomes derived from miR-30a-5p-inhibitor/RBE cells obviously diminished metastatic foci in lung and liver. By contrast, mice with miR-30a-5p-mimic/HCCC9810 exosomes administration increased lung, liver metastasis and induced more new vessels in liver under normoxic condition, with more apparent effect observed in mice with hypoxia-induced HCCC9810-originated exosomes treatment (Fig. 4O, 4P; Fig. S8A, 8B).

To further determine the critical role of exosomal miR-30a-5p in endothelium leakiness, we transfected miR-30a-5p-mimic and the corresponding NC-mimic into NC-mimic/HCCC9810-released exosomes, and miR-30a-5p-inhibitor and the corresponding NC-inhibitor into miR-30a-5p-mimic/HCCC9810-derived exosomes. Notably, endothelium angiogenesis, sprouts and cellular permeability was induced when HuVECs was treated with NC-mimic/HCCC9810 exosomes transfected with miR-30a-5p-mimic. However, miR-30a-5p-mimic/HCCC9810 exosomes with miR-30a-5p-inhibitor transfection treatment reversed the enhanced cellular capacity in angiogenesis, sprouts and permeability both in vivo and in vitro (Fig. S9A-9F). Cellular invasion assay revealed the same results (Fig. S9G). In addition, mice pretreated with NC-mimic/HCCC9810 exosomes with miR-30a-5p-mimic transfection increased metastatic foci in lung and liver. However, metastatic event and induced new vessels were obviously diminished in mice pretreated with miR-30a-5p-mimic/HCCC9810 exosomes with miR-30a-5p-inhibitor transfection (Fig. S9H, 9I). Combined, the findings above demonstrated that exosomal miR-30a-5p play an essential role in inducing vascular permeability and subsequent cancer invasion, metastasis.

### PDCD10 is the target of miR-30a-5p

**Figure 5:**
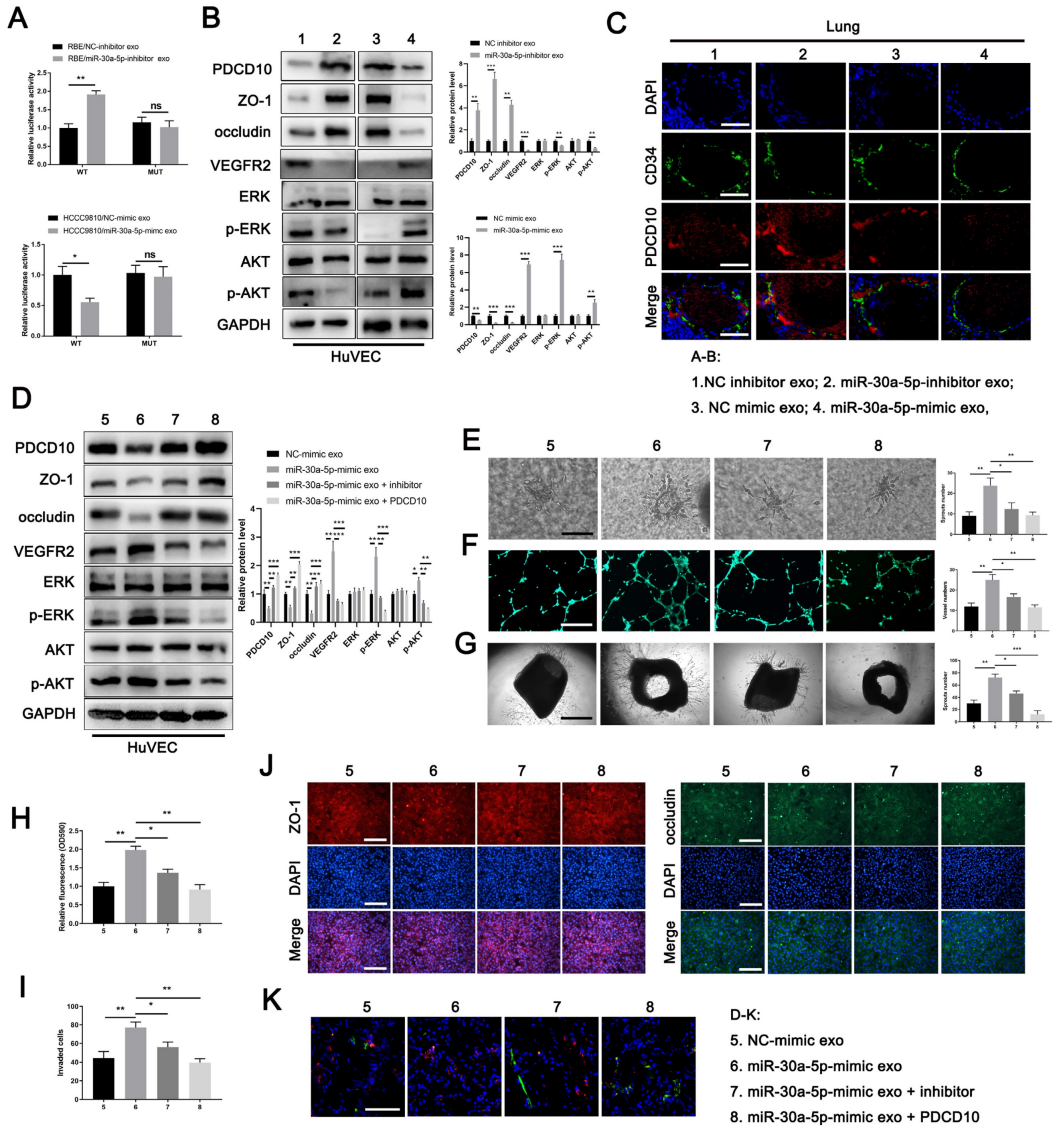
** MiR-30a-5p induced angiogenesis and vascular permeability by targeting PDCD10 A** Luciferase activities of 3'-UTR PDCD10-luc was examined in HUVECs cells after incubation with exosomes derived from RBE/miR-30a-5p-inhibitor, HCCC9810/miR-30a-5p-mimic and their corresponding control groups. **B** Protein level of PDCD10, ZO-1, occluding, VEGFR2 and downstream pathways including ERK and AKT were detected by western blotting in HuVECs cells with RBE/miR-30a-5p-inhibitor exosomes, HCCC9810/miR-30a-5p-mimic exosomes and their corresponding control exosomes treatment. **C** Mice were injected with exosomes mentioned above. Then, in vivo permeability assay was performed to detect the PDCD10 expression in endothelial of mice lung. **D** Exosomes from HCCC9810/miR-30a-5p-mimic and the corresponding normal control were obtained and transfected with miR-30a-5p inhibitor or PDCD10 plasmids. Then, proteins level of PDCD10, ZO-1, occluding, VEGFR2 and downstream pathways including ERK and AKT were detected by western blotting in HuVECs cells with these exosomes pretreatment. **E** 3D sprouts, **F** tube formation, **G** aortic ring, **H** permeability of the HUVECs monolayers to rhodamine, **I** the ability of RBE cell to invade HuVECs monolayers** J** immunofluorescence for ZO-1 and occluding, and **K** in vivo permeability assays were performed to detect the effects of exosomes mentioned above on angiogenesis and cellular permeability. *P < 0.05, **P < 0.01.

Previously, we reported that miR-30a-5p exerts oncogenic role in CCA by silencing SOCS3 expression based on online biological database 18. By further exploration, we detected that programmed cell death 10 (PDCD10), an apoptosis-related gene suppressing endothelial proliferation, migration, VEGFR2 expression and sustaining normal microvascular structure and barrier function 25, 26, is another target of miR-30a-5p (Fig. S10A). PDCD10 absence in the tumor-infiltrating endothelial has reported to activate glioblastoma cells and promote tumor growth 27. Notably, we detected that PDCD10 was negatively associated with miR-30a-5p expression in ICCA tissues (Fig. S10B). Based on TargetScan database, we observed the binding site between miR-30a-5p and PDCD10, and designed a mutated site in PDCD10 3'UTR for further verification (Fig. S10C). Luciferase reporter assay showed that the luciferase activity of reporter containing PDCD10 wild-type (WT) 3'-UTR rather than mutated (MUT) 3'-UTR in both 293T cell and HuVECs was evidently elevated after transfecting miR-30a-5p-inhibitor, but suppressed with miR-30a-5p-mimic transfection (Fig. S10D, S10E). Consistently, luciferase activity of PDCD10 WT 3'-UTR was strikingly increased in HuVECs with miR-30a-5p-inhibitor/RBE exosomes treatment but decreased when HuVECs incubated with exosomes released from miR-30a-5p-mimic/HCCC9810 (Fig. 5A). These results revealed that miR-30a-5p could target and silence PDCD10.

### Exosomal miR-30a-5p derived from ICCA promoted endothelium angiogenesis and induces vascular permeability dependent on PDCD10

To further clarify the effect of miR-30a-5p/PDCD10 axis in endothelium, we first transfected miR-30a-5p or PDCD10 into HuVECs and detect the alteration of phenotype in HuVECs. Western blotting showed that protein level of PDCD10, ZO-1 and occludin were decreased while VEGFR2 and downstream pathways including ERK and AKT were increased or activated with miR-30a-5p mimic transfection in HuVECs. However, the effects were partly reversed in HuVECs when co-transfected with miR-30a-5p mimic and PDCD10 plamid simultaneously (Fig. S10F). In line with that, miR-30a-5p-overexpressing HuVECs facilitated endothelial sprout and tube formation, induced cellular permeability and impaired endothelial barrier, whereas the effects were partly restored in HuVECs with PDCD10 and miR-30a-5p co-transfection (Fig. S10G-S10L). Subsequently, we transfected miR-30a-5p inhibitor into HuVECs. The expression level of PDCD10, ZO-1 and occluding were increased whereas VEGFR2 and downstream pathways including ERK and AKT were decreased or inactivated with miR-30a-5p silencing in HuVECs (Fig. S10M). Functional studies showed that miR-30a-5p silencing in HuVECs diminished endothelial sprouts and tube formation, suppressed permeability and enhanced cellular integrity (Fig. S10N-S10S). Together, these findings suggested that miR-30a-5p in endothelium induced angiogenesis and vascular permeability dependent on PDCD10.

To further detect the relationship between exosomal miR-30a-5p and endothelium-originated PDCD10, we treated HuVECs with miR-30a-5p-mimic/HCCC9810 exosomes, miR-30a-5p-inhibitor/RBE exosomes and their corresponding control exosomes. We found that the expression level of PDCD10 in HuVECs was increased with the treatment of miR-30a-5p-inhibitor/RBE exosomes. By contrast, decreased PDCD10 level was observed in HuVECs after miR-30a-5p-inhibitor/RBE exosomes incubation (Fig. 5B). Consistently, ZO-1 and occludin were increased while VEGFR2 and downstream pathways, including ERK and AKT were decreased or inactivated with miR-30a-5p-inhibitor/RBE exosomes treatment. MiR-30a-5p-mimic/HCCC9810 exosomes treatment led to the inverse effects (Fig. 5B). In addition, in vivo lung permeability model showed that PDCD10 expression was induced in mice pulmonary vessels whereas the effect was abrogated when mice were pretreated with miR-30a-5p-inhibitor/RBE exosomes (Fig. 5C). These results suggest that ICCA-derived exosomal miR-30a-5p could target and silence PDCD10 in endothelium.

To further probe the role of exosomal miR-30a-5p and PDCD10 in angiogenesis and vascular permeability, we obtained miR-30a-5p-mimic/HCCC9810 exosomes and the corresponding normal control exosomes. Part of miR-30a-5p-mimic/HCCC9810 exosomes was transfected with miR-30a-5p inhibitor or PDCD10 plasmids. We found that miR-30a-5p-mimic/HCCC9810 exosomes treatment suppressed PDCD10, ZO-1 and occluding expression, augmented VEGFR2 expression and ERK, AKT activation in HuVECs. However, miR-30a-5p-mimic/HCCC9810 exosomes transfected with miR-30a-5p inhibitor or PDCD10 plasmids had rescues effect in HuVECs (Fig. 5D). Functional studies demonstrated that miR-30a-5p-mimic/HCCC9810 exosomes incubation with HuVECs facilitated endothelial migration, sprouts and induced cellular permeability, whereas opposite phenomenon was observed in HuVECs administrated of miR-30a-5p-mimic/HCCC9810 exosomes transfected with miR-30a-5p inhibitor or PDCD10 plasmids (Fig. 5E-5K). The in vivo lung permeability assay showed the same effect (Fig. 5N). Combined, we clarified that CCA-derived exosomal miR-30a-5p could induce endothelium angiogenesis and vascular permeability dependent on PDCD10.

### EIF4B modulates packing of miR-30a-5p into exosomes

**Figure 6:**
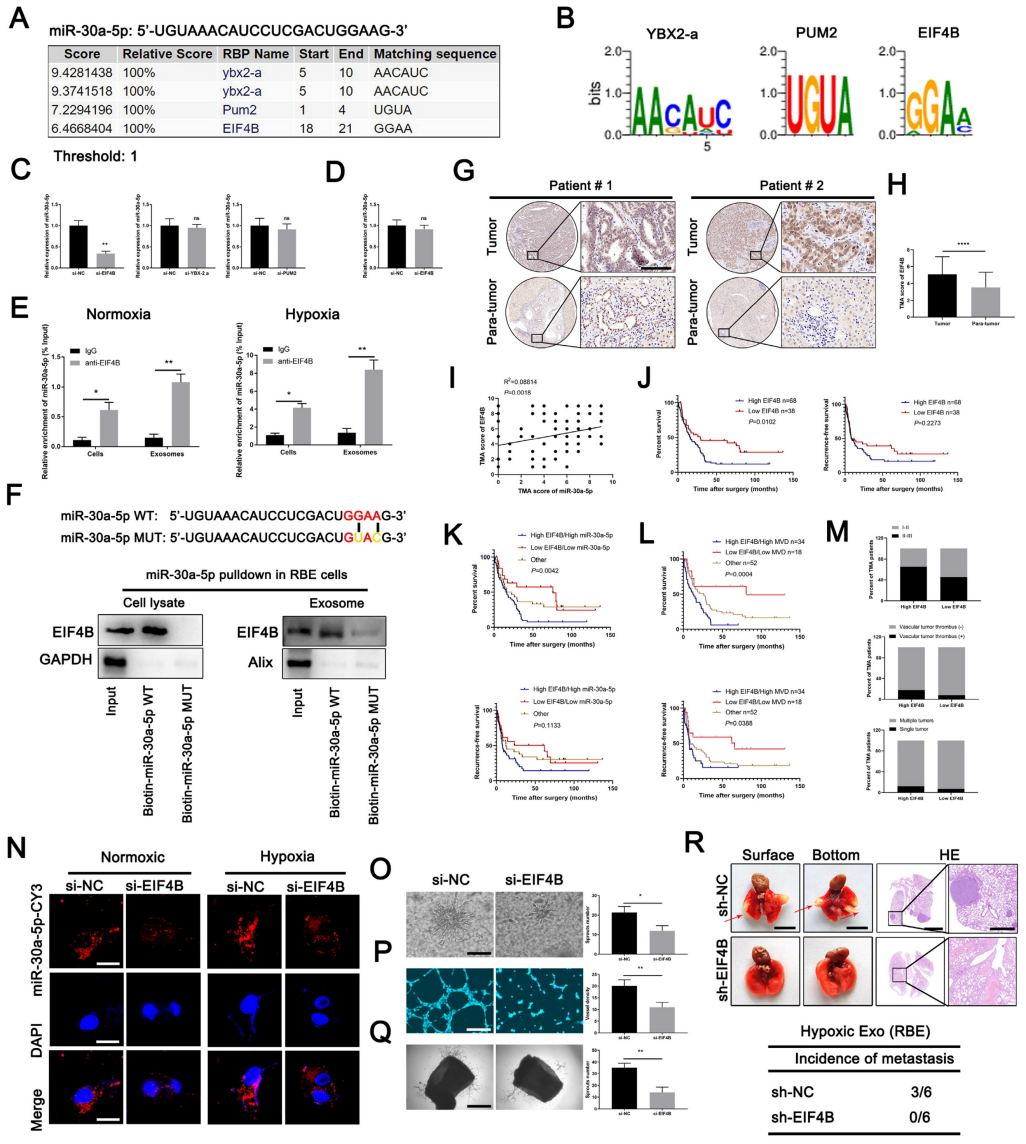
** EIF4B modulated packing of miR-30a-5p into exosomes. A, B** A specific interaction between the miR-30a-5p sequence and RBP motifs was predicted based on RBPDB analysis.** C** Exosomal miR-30a-5p expression secreted from CCA cells with YBX2-a, PUM2 and EIF4B silencing was detected by qRT-PCR. **D** MiR-30a-5p expression level in CCA cells with EIF4B silencing was detected by qRT-PCR. **E** RIP assays with anti-EIF4B antibody (IgG used for control) were performed on the cell or exosomal lysates from CCA cells under nomoxic or hypoxic condition. MiR-30a-5p levels in immunoprecipitated samples were determined by qRT-PCR and were calculated as percentages in respect to the input sample (% input). **F** MiR-30a-5p pulldown assay was performed in RBE cell lysate and the corresponding exosomes, then the enriched proteins were detected by western blotting. **G H** Representative images of distinct intensity of EIF4B expression based on IHC and the score of high/low EIF4B level in tumor and their corresponding para-tumor tissues were shown, the scores were calculated by intensity and percentage of stained cells. **I** The correlation of EIF4B and miR-30a-5p was analyzed based on IHC.** J** Kaplan-Meier curves for OS and RFS of ICCA patients with high/low EIF4B was shown. **K L** Kaplan-Meier curves for OS and RFS of ICCA patients combined with miR-30a-5p/EIF4B and MVD/EIF4B was shown. **M** Parameter analysis of ICCA patients with high/low EIF4B based on TMA IHC was shown. **N** CCA cells were transfected with miR-30a-5p-CY3 and subjected to nomoxia or hypoxia, then CCA cells-released exosomes were harvested and incubated with HuVECs for 48h. Fluorescence microscopy was used to detect red fluorescent signals in HuVECs. **O** 3D sprouts, **P** tube formation and **Q** aortic ring assays were performed to probe the effect of EIF4B on angiogenesis. **R** The effect of EIF4B on metastasis was analyzed based on lung metastasis model. *P < 0.05, **P < 0.01, ***P < 0.001.

By binding specific motifs, RNA binding proteins (RBPs) could exert effect on exosomal miRNA export and loading 28. We next investigated the potential RBPs for miR-30a-5p based on online database RBPDB (http://rbpdb.ccbr.utoronto.ca/). YBX2-A, PUM2 and EIF4B were identified as top three RBPs binding to miR-30a-5p with 100% threshold (Fig. 6A, 6B). To clarify which RBP was responsible for the interaction, we silenced YBX2-A, PUM2 and EIF4B in RBE cells (Fig. S11A, 11B) and detected that only EIF4B knockdown decreased miR-30a-5p level in CCA-derived exosomes, implying EIF4B facilitated the loading of miR-30a-5p into exosomes (Fig. 6C). However, miR-30a-5p expression level in RBE cells remained unchanged with EIF4B silencing (Fig. 6D). Subsequently, we performed RNA immunoprecipitation (RIP) assays in lysates from RBE cells and corresponding exosomes and detected a significant enrichment of miR-30a-5p in anti-EIF4B group as compared to anti-IgG group in both RBE cells and exosomes under normoxia or hypoxia, with more evident effect observed in cells and exosomes under hypoxia (Fig. 6E). Consistently, EIF4B was detectable in miR-30a-5p WT pulldown products rather than in that of miR-30a-5p MUT (Fig. 6F), further supporting that miR-30a-5p was a direct binding target of EIF4B. In addition, we observed that upregulated EIF4B in ICCA was significantly correlated with miR-30a-5p (Fig. 6G-6I; Fig. S11C-11F). Kaplan-Meier survival analysis showed that ICCA patients with high-EIF4B level correlated with reduced OS and RFS (Fig. 6J). Further combined analysis showed that ICCA patients with low EIF4B/low miR-30a-5p or low EIF4B/low MVD indicated better survival as compared to the others (Fig. 6K, 6L). Clinical parameters analysis showed that high-EIF4B ICCA patients tended to be accompanied by advanced stage, vascular tumor thrombus and multiple intrahepatic foci (Figure 6M). These data demonstrated that EIF4B acts as an oncogenic role inducing CCA progression.

Subsequent co-incubation assay showed that the transfer of RBE-derived exosomal miR-30a-5p to endothelium was apparently decreased after EIF4B silencing in CCA cells, regardless of hypoxia or normxia (Fig. 6N), suggesting EIF4B play a critical role in mediating miR-30a-5p packing into exosomes. Further examination indicated that EIF4B knockdown inhibited angiogenesis, sprout and diminished tumor growth and lung metastasis in mice (Fig. 6O-6R; Fig. S11G-11I). Together, these data demonstrate that oncoprotein EIF4B in ICCA mediates miR-30a-5p loading into exosome, induces angiogenesis and facilitated subsequent metastasis.

### Targeting miR-30a-5p improves the antiangiogenic efficacy of apatinib in CCA

**Figure 7:**
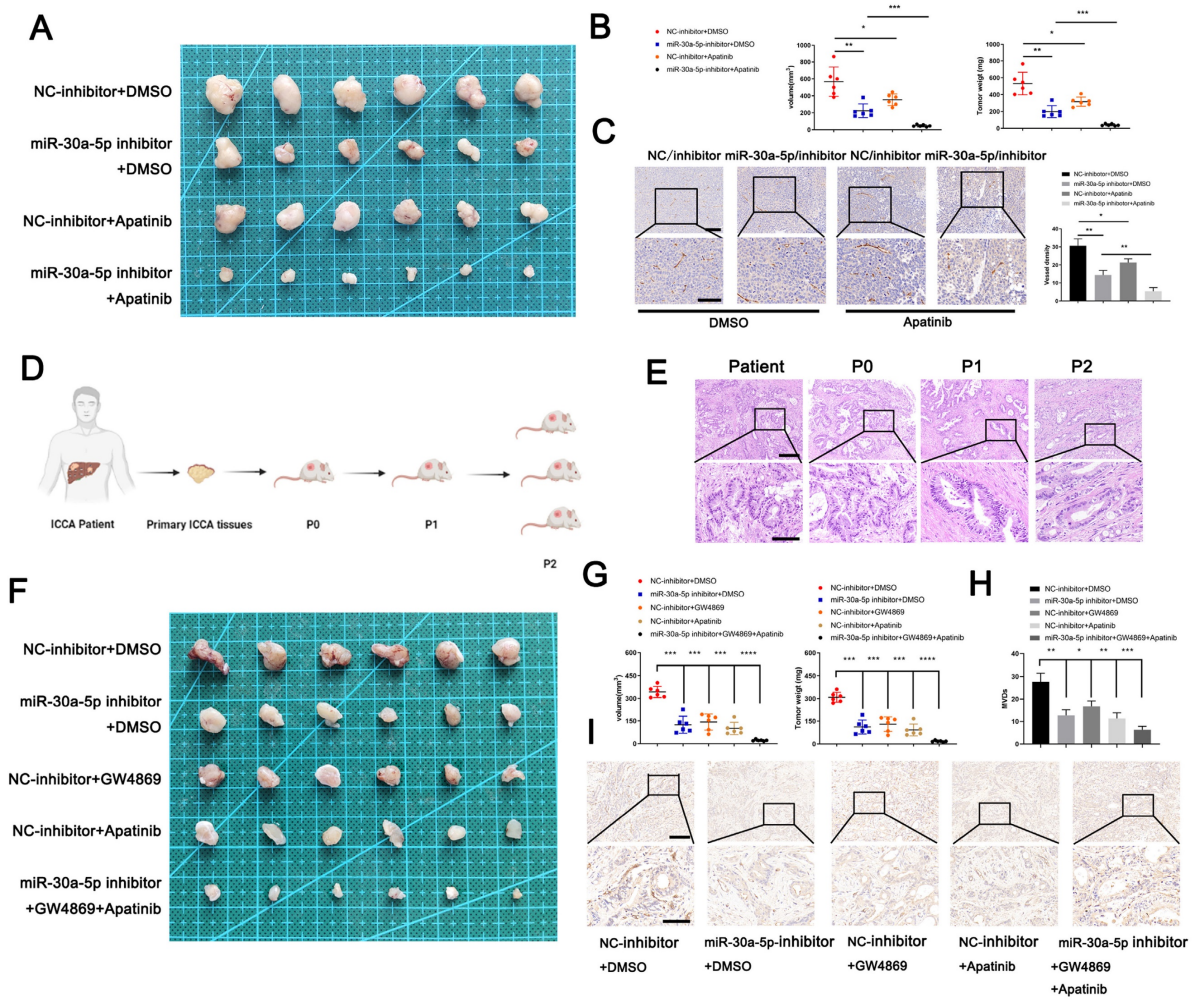
** Targeting miR-30a-5p in ICCA augmented the anti-angiogenic efficacy of apatinib. A** The efficacy of apatinib on ICCA was determined by xenografts model, the representative image of the tumors was shown.** B** The tumors removed from mice with apatinib treatment or not were quantified and weighted. **C** IHC for CD31 was carried out in removed tumors. **D** Illustration of the PDTX model construction. **E** H&E staining for tumor tissues from ICCA patient, P0, P1, and P2 was performed to confirm the same tissues source. **F** The efficacy of apatinib and targeting miR-30a-5p on ICCA was determined by PDTX model, the representative image of the tumors was shown. **G** The tumors removed from P2 mice treated with exosome inhibitor GW4869, miR-30a-5p inhibitor, apatinib, or not were quantified and weighted. **H** IHC for CD31 was carried out in tumors removed from P2 mice. *P < 0.05, **P < 0.01, ***P < 0.001.

To examine the clinical translation value of miR-30a-5p for ICCA patients, we first constructed xenograft model and probe the efficacy of a classical anti-angiogenesis drug, apatinib with different miR-30a-5p level. Apparently, apatinib treatment inhibited CCA growth in both groups when compared with those without apatinib treatment. However, apatinib-induced effects were more significant in mice with miR-30a-5p-silencing (Fig. 7A, 7B). Consistent results were observed in MVDs infiltration (Fig. 7C). Based on that, we subsequently utilized PDTX model of which conditions are closer to patients in nude mice to further examine the synergistical efficacy of apatinib and targeting miR-30a-5p. We first confirmed the successful model based on the same structural features by HE staining (Fig. 7D, 7E). After respective or combined treatment with miR-30a-5p-inhibitor, exosomes inhibitor GW4869 or apatinib, we removed the tumors from P2 mice. Obviously, tumor size and weight were decreased when mice were administrated of miR-30a-5p-inhibitor, exosomes inhibitor GW4869 or apatinib respectively. Furthermore, more significant efficacy was observed when the three were combined used (Fig. 7F, 7G). IHC staining for CD31 showed that density of new vessels was significantly decreased in tumors after treatment with monotherapy, with more remarkable trend detected in mice with combined therapy (Fig. 7H, 7I). These results suggest that combined application of targeting miR-30a-5p and apatinib could synergistically improve antiangiogenic efficacy in ICCA.

## Discussion

Being a complex and continuously evolving system that can be sculpted by cancer cells, TME has been proved to affect cancer development and progression by controlling the production of various growth factors, chemokines and other cytokines 29. TME is mainly constituted of extracellular matrix (ECM) and surrounding multiple cell population, including immune cells, fibroblasts and endothelial cells 8. In our study, we identified miR-30a-5p as a critical molecule correlating with the infiltration of vascular endothelial cells in ICCA, which prompted us to determine the role of miR-30a-5p in mediating TME crosstalk between cancer cells and endothelial cells. We revealed that miR-30a-5p facilitated cancer-associated angiogenesis and elevated vascular permeability in ICCA, suggesting that miR-30a-5p played a new role in modulating ICCA progression.

Tumor-associated neovascularization greatly facilitates tumor growth, hematogenous metastasis and postoperative recurrence 30. As one of the most common causes for tumor-associated angiogenesis, hypoxia, featured by driving gene dysregulation and disease progression, sustains in almost all stages of solid cancers 31. In respond to hypoxic stress, cancer cells hinder HIF-1α degradation, initiate transcription of targeted genes and recruit the infiltration of endothelial cells and induce subsequent angiogenesis 23, 31. In current study, we first clarified that hypoxia-induced HIF-1α in ICCA could bind and transcriptionally elevate miR-30a-5p level. In addition, we first demonstrated that ICCA patients with higher miR-30a-5p level have worse survival and suffer more frequently from recurrence, implying that HIF-1α induced miR-30a-5p in ICCA could be a promising indicator for prognosis analysis.

Exosome is excellent vehicle carrying diverse biomolecules including lipids, proteins, and nucleic acids and mediating TME reprogramming 32. Published studies have reported that miR-30a-5p could be loaded into exosomes that involved in malignant progression in lung adenocarcinoma, primary nephrotic syndrome in children and acute ischemic stroke in early-stage 33-35. Considered that miR-30a-5p was detectable in the peripheral blood of CCA patients on the basis of GEO database, we therefore hypothesized that miR-30a-5p might be harbored in ICCA-derived exosomes and involved in TME reprogramming. In line with that, our study confirmed the loading of miR-30a-5p in exosomes secreted from ICCA patients' peripheral blood and cell lines, and verified the enhanced effects of exosomal miR-30a-5p on inducing angiogenesis and vascular permeability. In addition, our data revealed that miR-30a-5p expression level in ICCA tissues was positively associated with that loaded in ICCA patients' serum-derived exosomes. Given that double-membrane exosomes shield the loadings from clearance by immune system 32, therefore, blood-derived miR-30a-5p might be a competent indicator in CCA diagnosis and monitoring for post-operation recurrence. Even though Tao et al. reported that vascular endothelial cell-derived exosomal miR-30a-5p inhibits lung adenocarcinoma malignant progression 35, however, few changes were observed in malignant behaviors of CCA cells treated with miR-30a-5p mimic/HuVECs-derived exosomes in our study. That might be due to the different genes targeted by miR-30a-5p in certain cancer context. The in-depth mechanisms merit further exploration.

The quantity of exosomal content affects cell signaling and biological function in recipient cells. Previous findings revealed that RNAs packaging from cell to exosome are directed by various RBPs through a specific motif in different cancer context. For instance, hnRNPA1 in head and neck cancer (HNC) increases the loading of miR-196a into cancer-associated fibroblasts (CAFs)-derived exosomes in miR-196a's UAGGUA sequence dependent way 28. In gastric carcinoma, KHDRBS3 expressed in CAFs directs circ_0088300 package into CAFs-derived exosomes by specifically binding to 100-200 bp sequence 36. Additionally, packaging of lncARSR in renal cell carcinoma (RCC) into exosomes could be mediated by hnRNPA2B1 37. In agreement with that, our present study revealed that EIF4B in CCA cells bound to the GGAA sequence of miR-30a-5p whose loading into CCA cell-derived exosomes was enhanced in the presence of EIF4B, which greatly induces cancer-associated angiogenesis and metastasis.

MiRNAs play a crucial role in various biological processes by binding to mRNA 3'-UTR region and exert suppressive effects on targeted genes 38. In our research, we identified PDCD10 as a critical regulator targeted by miR-30a-5p. PDCD10, initially deemed as an apoptosis-related gene, controls multiple biological functions, including endothelial cells junctions, cell proliferation and angiogenesis in cerebral cavernous malformation (CCM), cognitive disability, and different types of cancers 39. However, PDCD10 is a multifaceted regulator in cancer due to its different subcellular localization and various protein interactor. For instance, downregulated PDCD10 in human glioblastoma multiforme (GBM) is accompanied by enhanced malignant phenotypes and activation of Akt pathway 40. However, elevated PDCD10 in ovarian cancer and breast cancer induces more malignant behaviors 41-43. Even that, more reports focus the effects of PDCD10 on inhibiting endothelial angiogenesis, decreasing vascular permeability and remodeling vascular structure 39. Coincident with that, the current study clarified that PDCD10 in endothelial cells impaired cancer-associated neovascularization and improved vascular permeability. By targeting PDCD10, miR-30a-5p thus enhanced cancer-associated angiogenesis, induced vascular permeability and boosted the subsequent metastasis. These results indicated that exosomal miR-30a-5p derived from ICCA promoted endothelium angiogenesis and induced vascular permeability dependent on PDCD10.

Although regimen of gemcitabine plus oxaliplatin is the standard option for advanced or unresectable biliary tract cancer (BTC) 44, gemcitabine/oxaliplatin-associated drug-resistance remains a giant challenge 45. Recently, the combined application of anti-angiogenic targeted drugs and immune checkpoint inhibitors (ICIs) provides the promising strategy for advanced, recurrent and chemo-refractory cancer patients, including ICCA 46-48. A single-center, phase Ⅱ study showed that the objective response rate (ORR) of advanced or unresectable ICCA patients reaches 32.3% after the application of combining anti-PD-1 and anti-angiogenesis therapy. Furthermore, the regimens combining all three treatments (immune checkpoint inhibitor anti-PD-1 antibody, tyrosine kinase inhibitor lenvatinib, and GEMOX chemotherapy) for pure advanced ICC achieved great breakthrough, with ORR reaching 80% 49. In addition, apatinib, combined with camrelizumab based on multicenter research in China, has yielded excellent antitumor activity in primary liver cancer patients 47, suggesting that anti-angiogenic therapy could be a competent option for advanced or unresectable ICCA. Considered that ICCA-secreted exosomal miR-30a-5p induced neovascularization in our study, we therefore set out to determine the effects of apatinib on ICCA with high or low miR-30a-5p level. Our data revealed that combined treatment of miR-30a-5p inhibitor, exosomes inhibitor and apatinib remarkably increased the anti-cancer efficacy in PDX model as compared to that administrated respectively, suggesting that miR-30a-5p-based anti-angiogenic therapy could be a competent option for ICCA patients. However, due to the limits that our in vivo translational experiment was performed in immunodeficient mice and that mice-derived ICCA cell lines are not available, the clinical value of the therapy combined miR-30a-5p inhibitor, apatinib and ICIs remains unclear, which merits to be further verified.

## Conclusion

The current study demonstrated that miR-30a-5p harbored in ICCA-derived exosomes induced angiogenesis, impaired vascular permeability and facilitated distant metastasis. MiR-30a-5p expression level in ICCA tissues was positively correlated with that in circulating exosomes. Moreover, therapeutic regimen combined anti-angiogenesis and targeting miR-30a-5p could yield better efficacy. These data imply that clinical blood test and target therapy for miR-30a-5p in ICCA patients might be a useful way for diagnosis, treatment and postoperative monitoring.

## Supplementary Material

Supplementary materials and methods, figures and tables.

## Figures and Tables

**Table 1:** Univariate and multivariate analyses for OS of ICCA patients.

Characteristic	OS	
	*P* value	HR (95% CI)
**Univariate analysis**		
Age (≥60 vs. <60)	0.941	0.985 (0.652-1.488)
Gender (male vs. female)	0.298	0.801 (0.528-1.216)
Tumor size (≥5cm vs. <5cm)	0.047	1.513 (1.006-2.274)
Tumor number (single vs. multiple)	0.495	1. 092 (0.848-1.406)
Lymph node metastasis (yes vs. no)	0.000	3.221 (2.004-5.177)
Perineural invasion (yes vs. no)	0.302	1.274 (0.804-2.019)
Vascular invasion (yes vs. no)	0.477	1.301 (0.630-2.687)
Vascular tumor thrombus (yes vs. no)	0.025	1.887 (1.083-3.289)
R0 resection (yes vs. no)	0.033	1.759 (1.047-2.955)
Metastasis (yes vs. no)	0.030	3.091 (1.112-8.589)
MiR-30a-5p expression level	0.027	1.728 (1.204-3.055)
**Multivariate analysis**		
Tumor size (≥5cm vs. <5cm)	0.079	1.499 (0.955-2.353)
Lymph node metastasis (yes vs. no)	0.000	2.883 (1.729-4.807)
MiR-30a-5p expression level	0.045	1.676 (1.013-2.774)

## Abbreviations

ICCA: intrahepatic cholangiocarcinoma
TME: tumor microenvironment
qRT-PCR: quantitative real-time polymerase chain reaction
MVD: microvascular density
EIF4B: eukaryotic translation initiation factor 4B
ISH: in situ hybridization
ISH: immunohistochemistry
PDTX: patient derived tumor xenografts
PDCD10: programmed cell death 10
VEGFs: vascular-endothelial growth factors
BTC: biliary tract cancer
ICIs: immune checkpoint inhibitors
CAFs: cancer-associated fibroblasts
ECM: extracellular matrix
CCM: cerebral cavernous malformation
HNC: head and neck cancer
RCC: renal cell carcinoma
