# Supplementary material for: Exosomal miR-30a-5p promoted intrahepatic cholangiocarcinoma progression by increasing angiogenesis and vascular permeability in PDCD10 dependent manner
Source: Int J Biol Sci. 2023 Aug 28;19(14):4571–87. doi: 10.7150/ijbs.83170 (PMC10535699; doi:10.7150/ijbs.83170)
Supplement: Supplementary file 1 — Supplementary materials and methods, figures and tables. [file ijbsv19p4571s1.pdf]

Exosomal miR-30a-5p promoted intrahepatic cholangiocarcinoma progression by increasing angiogenesis and vascular permeability dependent on PDCD10

Wangjie Jiang[1,2*], Xiaoli Shi[1,2,3*], Lizhu Sun[4*], Yaodong Zhang[1,2], Xiangxu Kong[1,2], Xiao Yang[1,2], Yongmei Yin[4#], Changxian Li[1,2#], Xiangcheng Li[1,2#]

[1] Hepatobiliary Center, The First Affiliated Hospital of Nanjing Medical University;

[2] Key Laboratory of Liver Transplantation, Chinese Academy of Medical Sciences; NHC Key Laboratory of Living Donor Liver Transplantation (Nanjing Medical University), Nanjing, Jiangsu Province, China;

[3] School of Medicine, Southeast University, Nanjing, Jiangsu Province, China;

[4] Department of Oncology, The First Affiliated Hospital of Nanjing Medical University, Nanjing, Jiangsu Province, China.

* These authors contributed equally to this work.

Table of contents:

Supplementary materials and methods
Supplementary tables
Supplementary Figures
Figure S 1
Figure S 2
Figure S 3
Figure S 4
Figure S 5
Figure S 6
Figure S 7
Figure S 8
Figure S 9
Figure S 10
Figure S 11

Supplementary materials and methods

Cell cultures and transfection

Human CCA cell lines, HuVECs and 293T cell line were obtained from the Cell Bank of Chinese Academy of Sciences (Shanghai, China). All the cell lines were cultured in Dulbecco's Modified Eagle Medium (DMEM; Gibco, Invitrogen Life Technologies, CA, USA) containing 10% FBS (FBS; Gibco) and 100 units/ml of penicillin/streptomycin (Gibco) except for HuVECs which were cultured in total ECM medium. All cells were sustained in a humidified atmosphere of 5% CO_2 at 37°C.

For cell transfection, miR-30a-5p mimic, inhibitor and stable lentivirus vectors were purchased from Genechem (Shanghai, China) as previously[1]. Si-RNAs and plasmids for PDCD10, HIF-1α were obtained from GenePharma (Shanghai, China). In addition, we used Exo-Fect Exosome Transfection Kit (System Biosciences) to load miR-30a-5p mimics/inhibitor and PDCD10 plasmid into exosomes.

RNA Extraction and Quantitative real-time PCR

For extraction of cell lines and tissues RNA, we used the FastPure Cell/Tissue Total RNA Isolation Kit (Vazyme, Nanjing, China) according to the manufacturer's instructions. RNA purity and concentration were verified based on NanoDrop ND2000 (Thermo Scientific Inc, Waltham, MA). Reverse transcription was performed using Primescript RT Reagent (Vazyme, China). RT-qPCR was conducted with standard SYBR Green PCR kit (Vazyme) on the basis of the Thermal Cycler Dice Detection System (ABI 7900; Life Technologies). β-Actin was used as the internal control, and the relative transcription level of target genes was calculated by the 2-ΔΔCt method. For exosomal RNA detection, 30 nM miR-39 (cel-miR-39, RiboBio, Guangzhou, China) was added to each exosome sample for normalization before qRT-PCR. The detailed primer sequences were provided in Supplement Table 1

Western blotting

We used RIPA reagent kit (Beyotime, Shanghai, China) supplemented with PMSF (Beyotime) to extract cells and tissue samples protein. Equal amounts of protein in each lane were added and separated by SDS-PAGE (often 10% or 12.5% sodium dodecyl sulfate-polyacrylamide gel electrophoresis). Then, the gel was transferred to nitrocellulose membranes, blocked with QuickBlock™ Blocking Buffer (Beyotime, China) for 15 minutes, incubated with primary antibody overnight at 4°C and with secondary antibody at room temperature for 2h. The exposed Proteins images were visualized with ECL Plus (EMD Millipore, Billarica, MA, USA).

In situ hybridization (ISH)

We used LNA™ microRNA ISH kit (Exiqon; Woburn, MA, USA) to detect the expression of miR-30a-5p in ICCA TMA. Briefly, tissues slides were treated with fresh xylene for 15 min, hydrated using ethanol solutions and 2× saline sodium citrate (SSC) for 1 min respectively and then incubated with Proteinase K solution at 37 °C for 20 min. After that, 20 μL miR-30a-5p probe was used to incubate slides overnight which were then washed with SSC the next day. Subsequently, anti-digoxigenin was added to the slides at 37 °C for 60 min in a humid box when finishing blocking. The positive image was visualized by digital pathological scanning equipment.

Immunohistochemical (IHC) and Immunofluorescence (IF) analysis

We performed IHC analysis for CCA tissues based on the standard protocols of Super PlusTM High Sensitive and Rapid Immunohistochemical Kit (Elabscience, Wuhan, China). Briefly, the slides were treated with Dewaxing/Antigen Retrieval Buffer and SP Reagent B Peroxidase Blocking Buffer respectively. After that, primary antibodies in SP Reagent G Antibody Dilution Buffer were added to the slides and incubated at 4 °C overnight. The next day, SP Reagent C Polyperoxidase anti-Rabbit/Mouse IgG was used to treat the slides which were then incubated with SP Reagent F Hematoxylin Staining Buffer. The positive images were observed under light microscope. For IF analysis, cells were fixed with 4% formaldehyde for 10 min, permeabilized with 0.5% Triton X-100/PBS for 30min and blocked with 10% Donkey serum albumin at room temperature for 1h. Immunostaining was performed by incubating with Alexa Fluor 595 or Fluor 488 conjugated secondary antibodies (Beyotime) at room temperature for 2h following to incubating with corresponding primary antibodies overnight at 4 °C. Finally, cells were treated with 2-(4-Amidinophenyl)-6-indolecarbamidine dihydrochloride (Beyotime) at room temperature for 30min. Fluorescence images were visualized by fluorescence microscopy (Leica Microsystems Imaging Solutions, Cambridge, UK).

Transwell and wound-healing assay

HuVECs were pretreated with conditioned culture or exosomes for 24h. For tranwell assay, 5×10^4 pretreated HuVECs were seeded in the upper chamber of 24-well plate with 300 µL serum-free F12K medium and 600 µL total F12K medium was placed in the lower chamber. 24h later, the migrated HuVECs were stained with 0.1% crystal violet (Beyotime) for 20 min. For wound healing assay, 5×10^5 HuVECs mentioned above were seeded in six-well plate. Then, sterile 1 ml pipette tip was used to generate scratch wound. 48h later, the healing wound area was recorded and the calculated as a healing percentage.

In vitro angiogenesis, permeability and endothelial-associated invasion assays

For tube formation assay, HuVECs were incubated with exosome or conditioned culture for 48h and then seeded in Matrigel-coated 24-well plate for 8h. After that, the tube-like structure was visualized and counted under light microscope. For in vitro permeability assay, 10^5 HuVECs were incubated with exosome or conditioned culture for 48h and then seeded the top well of the transwell filters (0.4-µm, BD Biosciences). Subsequently, rhodamine B (Sigma) was added to the top of the insert and the bottom-well medium was collected to detect the fluorescence absorption at 544 nm excitation and 590 nm emission. For endothelial-associated invasion assay, RBE cells with GFP labeling were added to 10^5 HuVECs mentioned above. The invaded GFP^+ RBE cells were detected by fluorescence microscopy (Leica).

RNA immunoprecipitation assay (RIP)

RNA Immunoprecipitation Kit (Geneseed, Guangzhou, China) RIP assay was used to performed the assay. In brief, cells or exosomes were harvested and lysated with lysis buffer containing protease inhibitors, RNase inhibitors at 4 °C. Then, 50 µL of the lysate was used for input group and the other was incubated with 2µg anti-EIF4B or anti-IgG antibody at 4 °C overnight. The next day, 200 µL protein A+G beads was added to the mixture which was incubated at 4 °C for 2h. After washing with

the corresponding buffer, the enriched RNA/protein complex was eluted and the RNA was purified for reverse transcription. Subsequent qRT-PCR was performed to examine the relative enrichment of EIF4B to miR-30a-5p.

Pull down assay

The lysates of CCA cells and its derived exosomes were incubated with synthetic single-stranded wild-type or mutated miR-30a-5p oligonucleotides (100 pmol) with a biotin modification attachment at the 5' end via a spacer arm at 4℃ overnight (Sigma-Aldrich, USA). The next day, agarose beads were added to and incubated with prior mixture at 4 °C for 4 h. After that, the beads were washed and boiled to separate the binding proteins of miR-30a-5p. Western blotting was performed to detect the binding partners.

Dual-luciferase reporter assay

For verification of inverse correlation between miR-30a-5p and PDCD10, plasmids containing wild-type (WT) and mutated (MUT) 3'-UTRs of PDCD10 were synthesized by Genephama (Shanghai, China) and co-transfected with miR-30a-5p mimic into HuVECs and 293T cells using Lipofectamine 3000 (Invitrogen) or co-incubated with exosomes. For transcription analysis, pcDNA-HIF-1α and promoter-Luciferase-Renilla reporter plasmids based on pGL3 were co-transfected with miR-30a-5p mimic into 293T cells. After 48 h transfection, we investigated the luciferase activity based on the Dual-Luciferase Reporter Assay System (Promega). We prepared three repeats for each sample, and the assay was repeated three times.

3-Dimensional sprouting assay

HuVECs was pre-cultured in U-shaped 96-well plates with total ECM medium containing 0.3% methylcellulose (Sigma-Aldrich) for 24 h to form spheroid. After that, spheroid HuVECs was cultured in ECM medium mixed with Matrigel® (1:1) which was then polymerized at 37°C before adding medium on the top of the mixture. The images of sprouted cells were detected at 2-day later using a microscope.

Artery ring

8-week-old Sprague Dawley (SD) rats were purchased from Weitonglihua Biotechnology. For aortic ring assay, 96-well plate was pre-coated with matrigel. Then, rat-derived thoracic aortas were obtained and cut into cross-sections rings which were subsequently placed on wells with ECM medium supplemented with 10% fetal bovine serum, 50 U/ml penicillin, 50 μg/ml streptomycin in 37 °C incubator. 24 h after that, the medium was replaced by conditioned medium, exosomes-containing medium or that containing miR-30a-5p mimics, miR-30a-5p inhibitor, or PDCD10 overexpressing plasmid for further study. The sprouts number was observed on day 5 with microscope.

Exosome isolation and verification

For exosomes isolation, CCA cells with 80% confluence were cultured in media supplemented with exosome-free serum for 48 h. Then, the conditioned media were collected and centrifuged at 500 g for 10 min and 10, 000 g for 30 min at 4 °C. After that, the supernatant was filtered through a 0.22μm PVDF membrane (Millipore, USA), and ultracentrifuged at 110, 000 g for 70 min at 4 °C. Exosomes

obtained from the pellet were resuspended in PBS and preserved at -80 °C. Exosomes were identified by transmission electron microscopy (TEM) (H-7500; Hitachi, Tokyo, Japan) based on negative staining.

For analysis of exosome quantification, NanoSight NS300 instrument (NTA, Malvern Instruments Ltd. UK) was used to track the size and density of the exosomes. Briefly, exosomes obtained by ultrafast centrifugation were resuspended with 300ul PBS, then further diluted with 10ml PBS for loading. The sample pool was rinsed with pure water, and then the sample was driven into the pool to obtain exosome diameter data. The diameter distribution of exosomes was treated with Rstuido software.

For exosome labelling, PKH67 (Sigma) was used to mark the exosomes following the instructions. Labelled exosomes were obtained by repeating the step mentioned above. 50 μg exosomes were incubated with 5×10^5 HuVECs cells for indicated times.

Mice models

Six-week-old male BALB/C nude mice were purchased from Weitonglihua Biotechnology and housed in a specific pathogen-free (SPF) environment. For in vivo permeability assay, mice were pretreated with PKH67-labeled exosome intravenously every other day for 2 weeks. Then, 100mg/kg rhodamine was intravenously injected into tail vein of nude mice 3 h before sacrificing by CO2 asphyxiation. The mice lungs were removed for fluorescence detection. For lung metastasis model, exosome-exposed mice were inoculated with 5×10^6 RBE and HCCC9810 cells in 100 μL PBS via tail vein. Eight weeks later, mice were sacrificed, and the lungs were obtained for observation and further examination. For liver metastasis model, exosome-exposed mice were injected with 5×10^6 RBE and HCCC9810 cells in 100 μL PBS through the spleen parenchyma. The spleen was removed after injection to avoid intrasplenic dissemination. Four weeks later, mice were sacrificed, and the livers were obtained for observation and further examination. Apatinib (50 mg/kg) was delivered by the method of oral gavage daily.

Patient derived tumor xenografts (PDTX) model

Fresh tumor tissues resected from ICCA patient was preserved in iced DMEM with 10% fetal bovine serum, cut into 2*2*2 mm^3 pieces and washed with PBS containing penicillin (500 U/mL) and streptomycin (500 μg/ mL) twice. Then, tissue pieces were subcutaneously transplanted into the backs of NSG mice (P0). All procedures were performed within 2 hours. For ICCA tumor passage, we harvested the xenografts tissues when the volume reached 1-2cm^3, repeated the aforementioned steps and transplanted pieces into nude mice. We performed subsequent experiments in P2 mice. For apatinib efficacy analysis, mice were divided into NC-inhibitor+DMSO group, NC-inhibitor+GW4869 group, miR-30a-5p-inhibitor+DMSO group, NC-inhibitor+ apatinib group and miR-30a-5p-inhibitor+GW4869+apatinib group. All drugs-associated treatment was carried out when tumor volume reached 50 mm^3. MiR-30a-5p inhibitor was intratumorally administrated continuously for 20 days. GW4869 (2 mg/kg) was intraperitoneally injected every 2 days. Apatinib (50 mg/kg) was delivered by the method of oral gavage daily.

All animal experiments were approved by the Institutional Animal Care and Use Committee at Nanjing Medical University (approval number: No. IACUC-2203004).

Reference

1. Zhang JW, Wang X, Li GC, Wang D, Han S, Zhang YD, Luo CH, Wang HW, Jiang WJ, Li CX, Li XC (2020) MiR-30a-5p promotes cholangiocarcinoma cell proliferation through targeting SOCS3. J Cancer 11 (12):3604-3614. doi:10.7150/jca.41437

Supplementary table 1. Antibodies

**Name**	**Supplier**	**Cat no.**	**Clone no.**
HIF-1α	Proteintech	20960-1-AP	
CD34	Abcam	ab81289	EP373Y
CD31	Abcam	ab9498	JC/70A
CD63	Abcam	ab134045	EPR5702
Alix	Abcam	ab275377	EPR23653-32
TSG101	Abcam	ab125011	EPR7130(B)
ZO-1	Proteintech	21773-1-AP	
occludin	Proteintech	27260-1-AP	
GAPDH	Proteintech	60004-1-Ig	
PDCD10	Proteintech	10294-2-AP	
VEGFR2	Abcam	ab2349	
ERK	Cell Signaling Technology	#4695	137F5
p-ERK	Cell Signaling Technology	#4370	D13.14.4E
AKT	Cell Signaling Technology	#4691	C67E7
p-AKT	Cell Signaling Technology	#4060	D9E
YBX2-α	Proteintech	13538-1-AP	
PUM2	Proteintech	11586-1-AP	
EIF4B	Proteintech	17917-1-AP	
HRP-linked anti-rabbit IgG	Cell Signaling Technology	#7074	
HRP-linked anti-mouse IgG	Cell Signaling Technology	#7076	
Alexa Fluor 594 donkey anti-rabbit IgG	Proteintech	SA00013-8	
Alexa Fluor 488 donkey anti-mouse IgG	Proteintech	SA00013-5	

Supplementary table 2. Sequence

**Name**	**Sequence**	**Supplier**
Primer: CD31 forward	AACAGTGTTGACATGAAGAGCC	Realgene
Primer: CD31 reverse	TGTAAAACAGCACGTCATCCTT	Realgene
Primer: CD34 forward	CTACAACACCTAGTACCCTTGGA	Realgene
Primer: CD34 reverse	GGTGAACACTGTGCTGATTACA	Realgene
Primer: GAPDH forward	GGAGCGAGATCCCTCCAAAAT	Realgene
Primer: GAPDH reverse	GGCTGTTGTCATACTTCTCATGG	Realgene

Primer: PDCD10 forward	GCCCCTCTATGCAGTCATGTA	Realgene
Primer: PDCD10 reverse	AGCCTTGATGAAAGCGGCTC	Realgene
Primer: EIF4B forward	AGGGGAAGACTATCTCCCTAACA	Realgene
Primer: EIF4B reverse	TCATCCGTTTCATCAGCCCAG	Realgene
Primer: YBX2-α forward	GCTGGCAATCCAAGTCCTG	Realgene
Primer: YBX2-α reverse	ACGTCGTAGTAACTTCAGGTCTC	Realgene
Primer: PUM2 forward	TCGGGGAATGGGAGAGCTTT	Realgene
Primer: PUM2 reverse	GCTGGGACATTGAATGGTGAGA	Realgene
Primer: MiR-30a-5p forward	CGCGATGTTGAAACATCCTCGAC	Realgene
Primer: MiR-39 forward	GGTCACCGGGTGTAAATCAGCTTG	Realgene
Primer: U6 forward	GGAACGATACAGAGAAGATTAGC	Realgene

Supplementary table 3. Regents

Name	Cat no.	Supplier
MiR-30a-5p-CY3		RiboBio Co
PKH67	PKH67GL	Sigma
GW4869	HY-19363	MCE
Rhodamine	83689	Sigma
Lipo3000	L3000015	Invitrogen

Supplementary figures

Figure S1

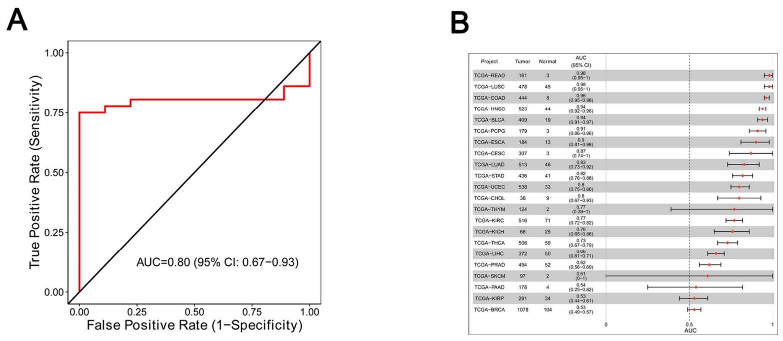

Figure S1 MiR-30a-5p was a critical regulator in CCA. The AUC value of miR-30a-5p in **A** CCA and **B** other cancer types was probed based on TCGA database.

Figure S2

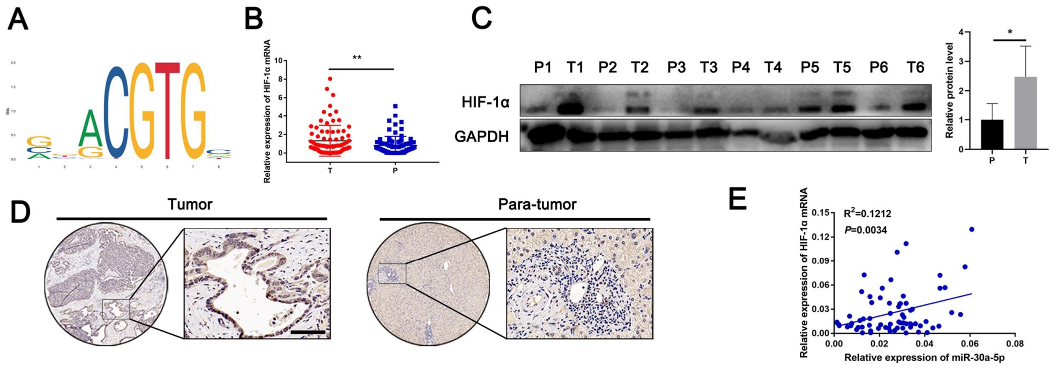

Figure S2 HIF-1α transcriptionally activated miR-30a-5p and was increased in ICCA. A The binding motif of HIF-1α was shown. **B** The relative expression of HIF-1α in sixty-nine pairs of ICCA was detected by qRT-PCR. **C** The expression level of HIF-1α in CCA tissues was examined by western blotting and **D** IHC. **E** The correlation between miR-30a-5p and HIF-1α in sixty-nine pairs of ICCA was analyzed based on qRT-PCR data. *$*P < 0.05$, $**P < 0.01$.*

Figure S3

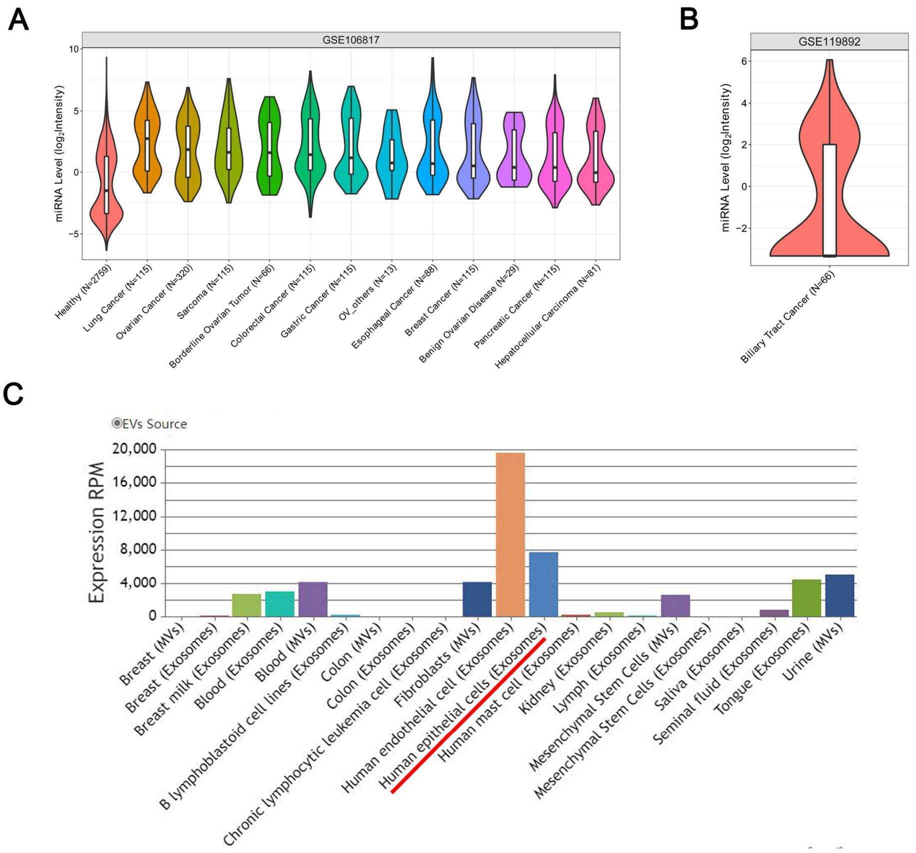

Figure S3 MiR-30a-5p could be loaded in exosomes. A Circulating miR-30a-5p expression in peripheral blood derived from multiple ranges of cancer patients was shown. **B** Circulating miR-30a-5p expression in peripheral blood derived from CCA patients was shown. **C** MiR-30a-5p could be released from different tissue and cell types.

Figure S4

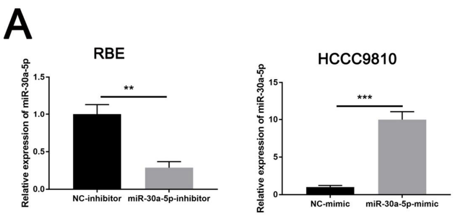

Figure S4 MiR-30a-5p was depleted or overexpressed in CCA cells. A The transfection efficiency of miR-30a-5p in RBE and HCCC9810 cells was confirmed by qRT-PCR. *$*P < 0.05$, $**P < 0.01$, $***P < 0.001$.*

Figure S5

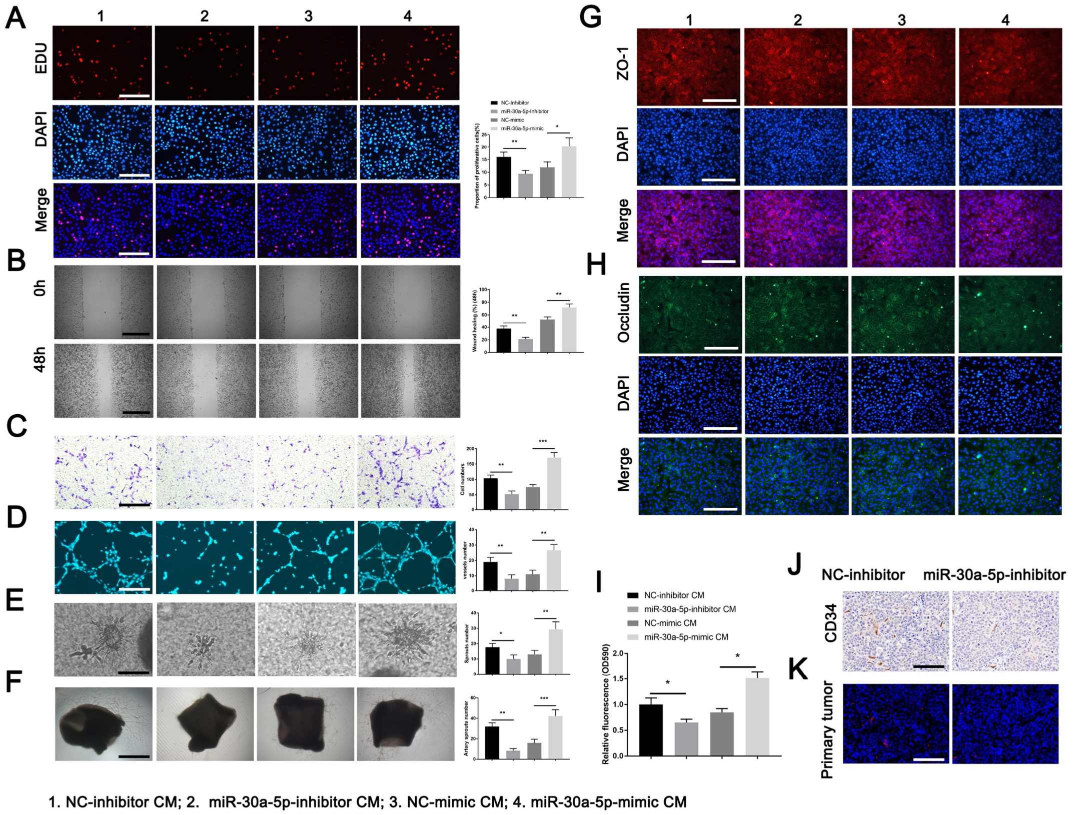

Figure S5 MiR-30a-5p facilitated endothelium proliferation migration, angiogenesis and induced vascular permeability. HuVECs were pretreated with culture medium (CM) from miR-30a-5p-overexpressing or miR-30a-5p-silencing cells, then the alteration of proliferation and migration was examined by **A** EDU, **B** wound-healing, **C** transwell, **D** tube formation, **E** 3D sprouts and **F** aortic ring assays. **G, H** Alteration of permeability was analyzed based on immunofluorescence analysis for tight junction related proteins including ZO-1 and occludin in HuVECs treated with the CM. **I** Permeability of the HUVECs monolayers pretreated with the CM to rhodamine was detected. **J** CD31 IHC staining was performed to examine miR-30a-5p-induced angiogenesis. **K** Rhodamine was intravenously injected into nude mice with RBE/NC-inhibitor or RBE/miR-30a-5p-inhibitor inoculation 2 hours before sacrifice, then tumors were removed for immunofluorescence to detect the alteration of vascular permeability. $*P < 0.05$, $**P < 0.01$.

Figure S6

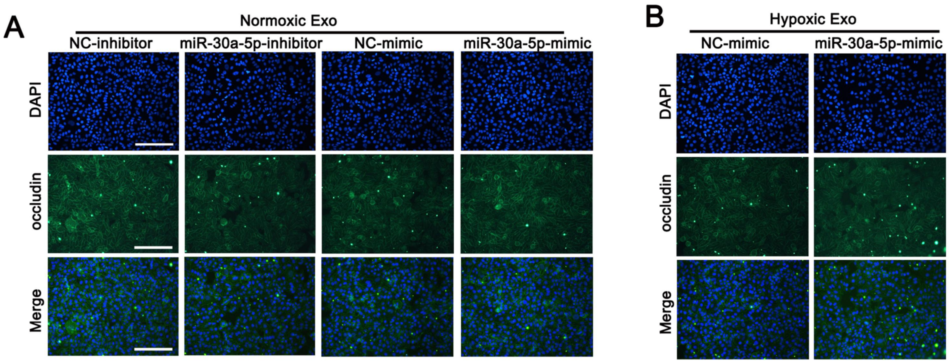

Figure S6 Exosomal miR-30a-5p released from CCA cells enhanced endothelial permeability. A, B The effects of RBE/miR-30a-5p-inhibitor and HCCC9810/miR-30a-5p-mimic released exosomes on endothelial permeability was explored by immunofluorescence for occludin.

Figure S7

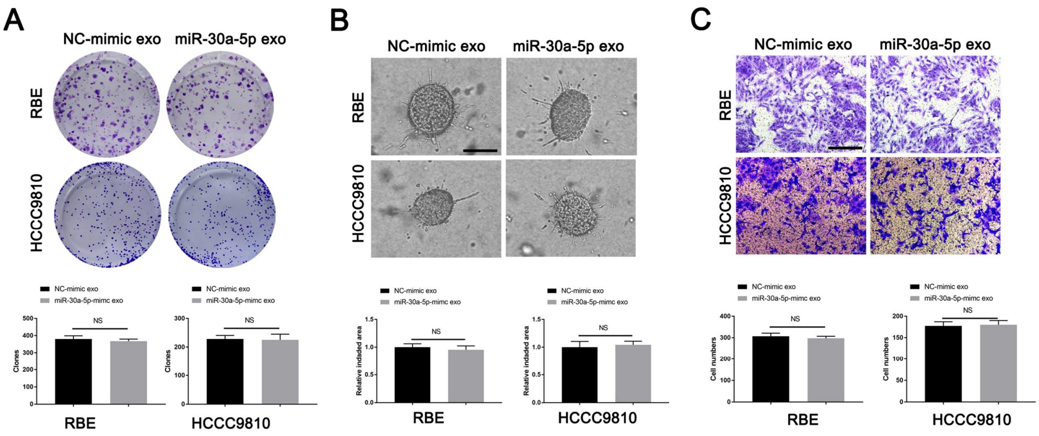

Figure S7 Endothelial-originated exosomal miR-30a-5p did not exert effects on CCA cells. A Colony formation, **B** 3D invasion and **C** transwell migration was performed to probe the effects of HuVECs-originated exosomal miR-30a-5p on CCA cells.

Figure S8

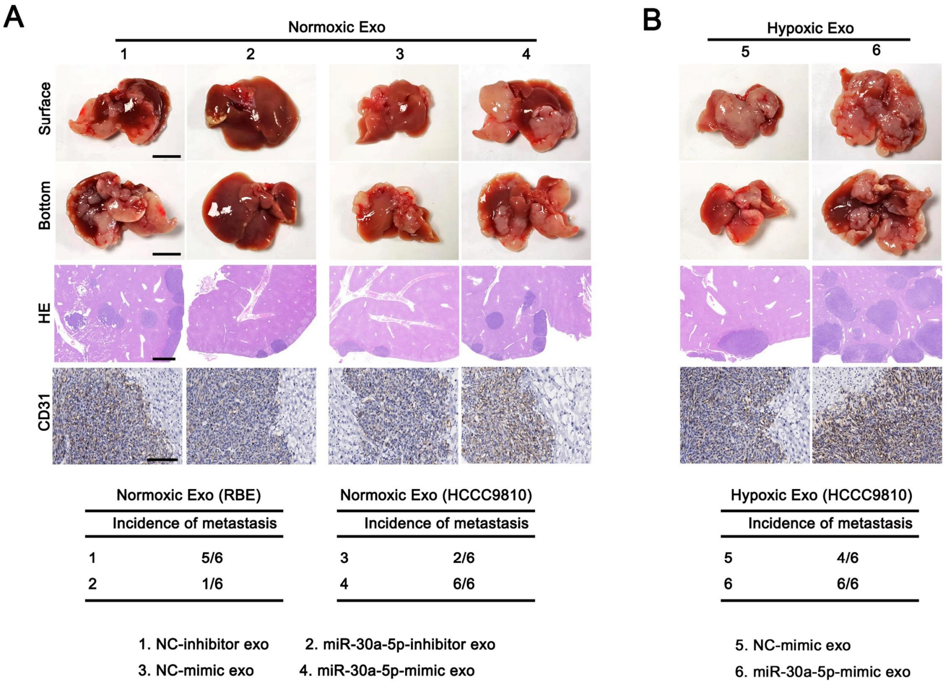

Figure S8 Exosomal miR-30a-5p induced liver metastasis. Mice were pretreated with 5 μg hypoxia/normoxia-treated exosomes via tail vein every other day for 2 weeks, then mice were subjected to the inoculation of RBE (group 1 and 2) or HCCC9810 (group 3-6) cells though spleen. The representative images shown the metastatic loci in liver and vascular density in liver metastasis model. **P < 0.05, **P < 0.01.*

Figure S9

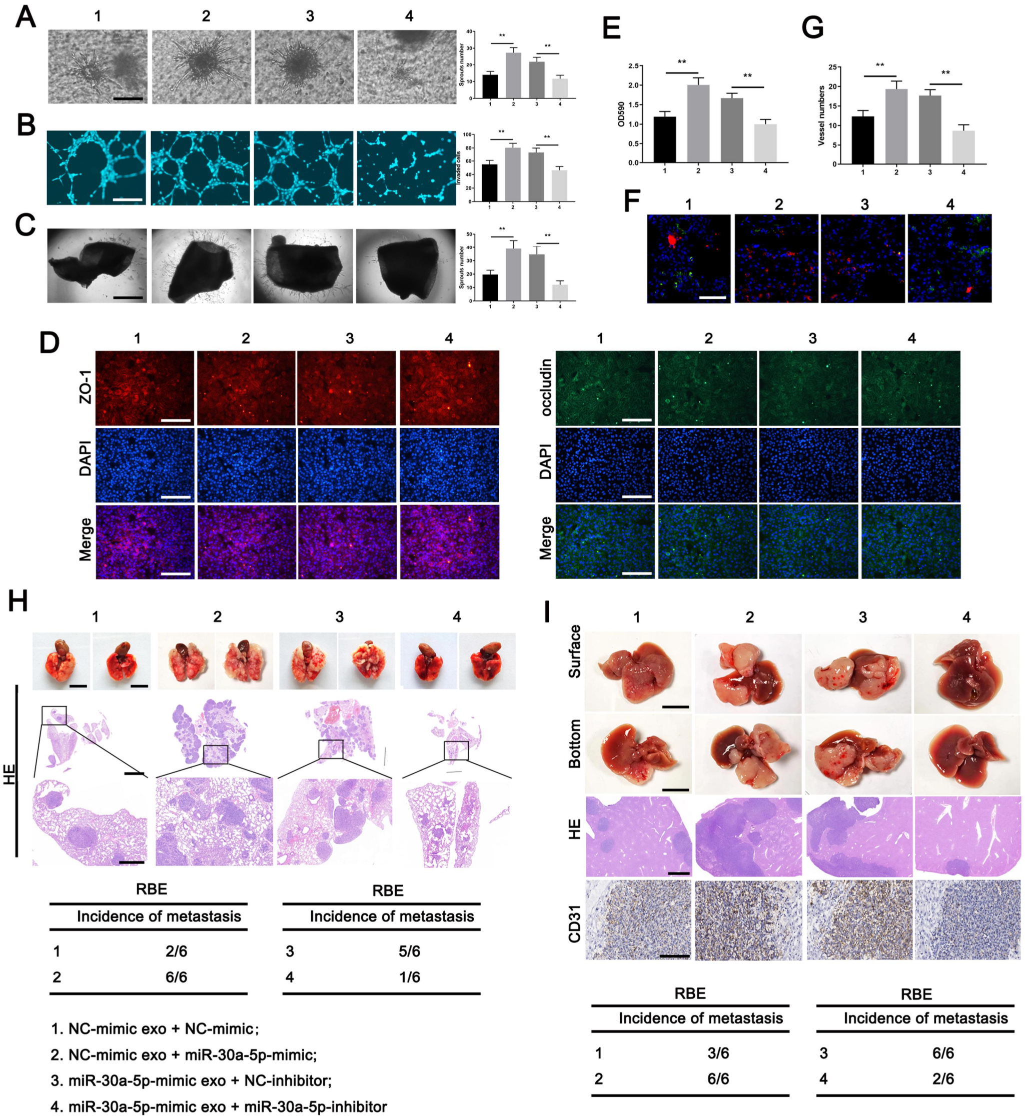

Figure S9 MiR-30a-5p induced vascular leakiness. Exosomes derived from HCCC9810/NC, HCCC9810/miR-30a-5p were transfected with miR-30a-5p mimics, miR-30a-5p inhibitor, respectively. Then, HuVECs was treated with these exosomes. **A** 3D sprouts, **B** tube formation, **C** aortic ring assays **D** immunofluorescence for ZO-1 and occluding, and **E** rhodamine-associated in vitro permeability assay and **F** in vivo permeability assay were performed to detect effects of the exosomes on angiogenesis and endothelial permeability. **G** The ability of RBE cell to invade HuVECs monolayers after treated with these exosomes was examined. Mice were pretreated with 5 μg of the exosomes via tail vein every other day for 2 weeks, then mice were subjected to the inoculation of HCCC9810 cells via tail vein or spleen. The representative images shown the metastatic loci in **H** lung or **I** liver and vascular density in **I** liver. *$*P < 0.05$, $**P < 0.01$.*

Figure S10

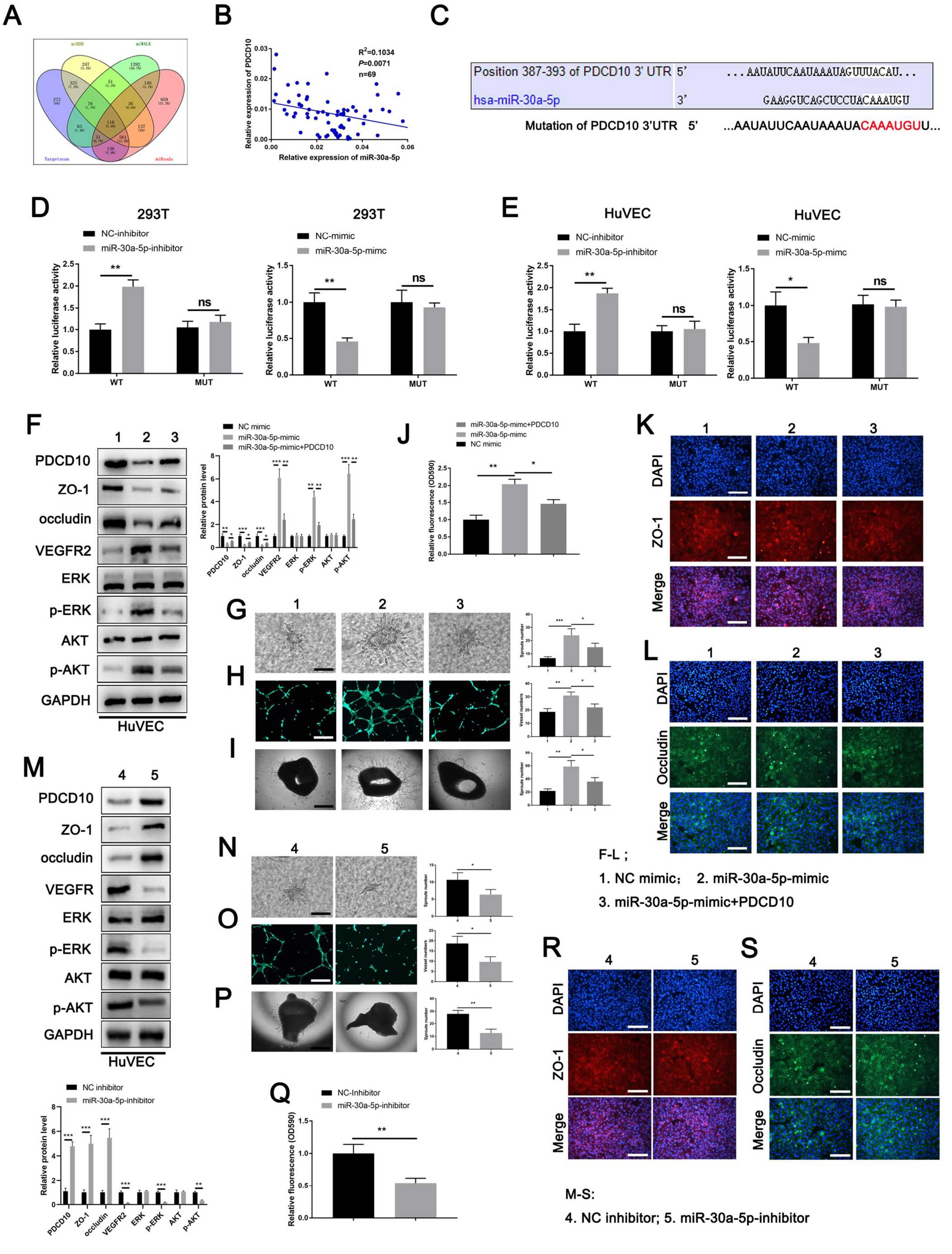

Figure S10 MiR-30a-5p induced angiogenesis and vascular permeability by targeting PDCD10 A The downstream molecules targeted by miR-30a-5p was predicted by online database. **B** The correlation between miR-30a-5p and PDCD10 was analyzed in ICCA tissues. **C** The binding site of miR-30a-5p and PDCD10 was predicted based on Targetscan database and a mutated binding site was designed. **D, E** Luciferase activities of 3'-UTR PDCD10-luc was examined in 293T and HUVECs cells after transfected with miR-30a-5p-inhibitor and miR-30a-5p-mimic. **F** Protein level of PDCD10, ZO-1, occluding, VEGFR2 and downstream pathways including ERK and AKT were detected by western blotting in HuVECs cells with miR-30a-5p mimic transfection or miR-30a-5p mimic/PDCD10 plamids

transfection simultaneously. **G** 3D sprouts, **H** tube formation and **I** aortic ring assays was performed in HuVECs cells transfected with miR-30a-5p mimic or miR-30a-5p mimic/PDCD10 plamids simultaneously. **J-L** HuVECs cells were transfected with miR-30a-5p mimic or miR-30a-5p mimic/PDCD10 plamids simultaneously, then, permeability assay was performed based on rhodamine and immunofluorescence for ZO-1, occluding. **M** Protein level of PDCD10, ZO-1, occluding, VEGFR2 and downstream pathways including ERK and AKT were detected by western blotting in HuVECs cells with miR-30a-5p inhibitor transfection. **N** 3D sprouts, **O** tube formation and **P** aortic ring assays was performed in HuVECs cells with miR-30a-5p inhibitor transfection. **Q** Assays including rhodamine and **R, S** immunofluorescence for ZO-1, occluding was performed to detect the alteration of permeability in HuVECs cells transfected with miR-30a-5p inhibitor. **P < 0.05, **P < 0.01.*

Figure S11

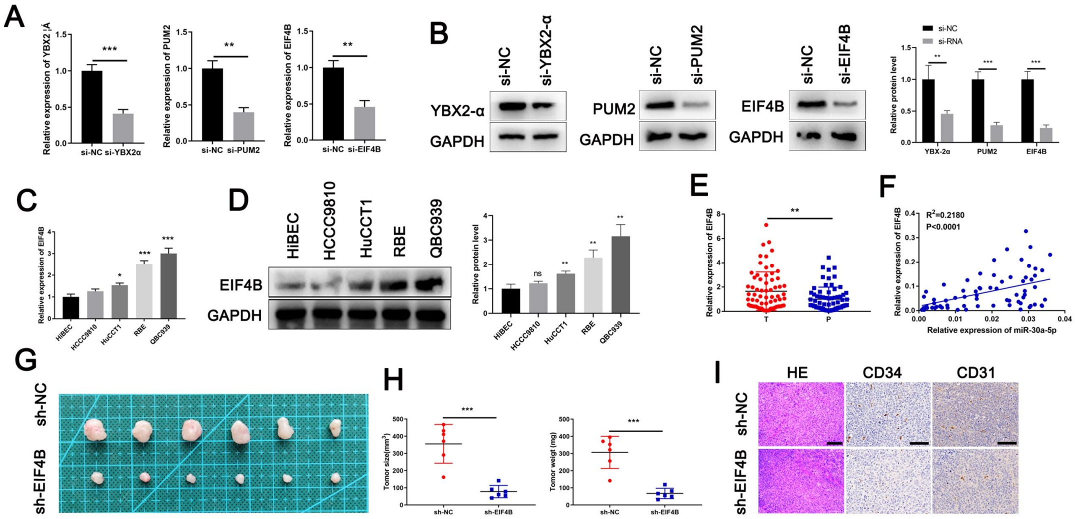

Figure S11 EIF4B modulated packing of miR-30a-5p into exosomes. YBX2-α, PUM2 and EIF4B were silenced and confirmed by **A** qRT-PCR and **B** western blotting. The expression level of EIF4B in CCA cell lines and normal bile duct epithelial was detected by **C** qRT-PCR and **D** western blotting. **E** The EIF4B mRNA expression level in 69 pairs of CCA samples and adjacent normal tissues was determined. **F** Correlation analysis was performed between miR-30a-5p expression and EIF4B expression in CCA tissues. **G** The effect of EIF4B in vivo was determined by xenografts model, the representative image of the tumors was shown. **H** The tumors removed from nude mice were quantified and weighted. **I** HE staining and IHC for vascular density (CD34 and CD31) were carried out in removed tumors. **P < 0.05, **P < 0.01, ***P < 0.001.*
